# MSEA: detection and quantification of mutation hotspots through mutation set enrichment analysis

**DOI:** 10.1186/s13059-014-0489-9

**Published:** 2014-10-28

**Authors:** Peilin Jia, Quan Wang, Qingxia Chen, Katherine E Hutchinson, William Pao, Zhongming Zhao

**Affiliations:** Department of Biomedical Informatics, Vanderbilt University School of Medicine, Nashville, TN 37203 USA; Center for Quantitative Sciences, Vanderbilt University Medical Center, Nashville, TN 37232 USA; Department of Biostatistics, Vanderbilt University School of Medicine, Nashville, TN 37232 USA; Department of Cancer Biology, Vanderbilt University School of Medicine, Nashville, TN 37232 USA; Department of Medicine/Division of Hematology-Oncology, Vanderbilt University School of Medicine, Nashville, TN 37232 USA; Vanderbilt-Ingram Cancer Center, Vanderbilt University School of Medicine, Nashville, TN 37232 USA

## Abstract

Many cancer genes form mutation hotspots that disrupt their functional domains or active sites, leading to gain- or loss-of-function. We propose a mutation set enrichment analysis (MSEA) implemented by two novel methods, MSEA-clust and MSEA-domain, to predict cancer genes based on mutation hotspot patterns. MSEA methods are evaluated by both simulated and real cancer data. We find approximately 51% of the eligible known cancer genes form detectable mutation hotspots. Application of MSEA in eight cancers reveals a total of 82 genes with mutation hotspots, including well-studied cancer genes, known cancer genes re-found in new cancer types, and novel cancer genes.

**Electronic supplementary material:**

The online version of this article (doi:10.1186/s13059-014-0489-9) contains supplementary material, which is available to authorized users.

## Background

Single nucleotide variants (SNVs) and short insertions and deletions (indels) are the most abundant somatic mutations in cancer genomes. Next-generation sequencing (NGS) studies have revealed that tens of thousands of SNVs and indels may exist in a cancer genome, yet many of them do not play important roles in tumorigenesis. Currently, one major challenge is to distinguish mutations that confer a selective advantage (so-called 'driver mutations') to cancer cells from those that do not offer such advantages ('passenger mutations') [1].

Traditionally, candidate cancer genes or mutations have been predicted by a frequency-based approach, where genes with many recurrent mutations are highly ranked [2]. However, this approach suffers from several known limitations, such as a high false positive rate, and it often misses low-frequency yet genuine cancer genes [3]. The recent explosion of NGS data has placed a strong demand on bioinformatics approaches for cancer gene prediction [4-8]. In general, current methods can be categorized into three groups. The first group consists of methods that assess sequence contexts, evolutionary conservation, and the functional impact of mutations. Representative methods of this group include SIFT [9], PolyPhen [4], and MutationAssessor [10]. Methods in the second group are mainly feature-oriented. These methods study and summarize common features from known cancer genes and mutations and propose data-mining algorithms to rank candidate genes that resemble the features observed in those known genes and mutations, such as OncodriveFM [11] and OncodriveCLUST [12]. The third group includes advanced pathway and network analyses. These methods take advantage of functional regulations in multi-dimensional -omics data, curated functional pathways and networks, and information regarding the complex regulations and interactions among proteins [6,13]. Representative methods of this group include DriverNet [8], HotNet [14], and MEMo [15]. In practice, each of the above groups of methods has its own advantages and shortcomings in detecting cancer genes or mutations with unique features [7,16].

Herein, we focus on mutation hotspot patterns in genes. Many driver mutations, especially nonsynonymous ones, recurrently occur in the functional regions of proteins (for example, kinase domains or binding domains) [17] or interrupt active sites (for example, phosphorylation sites) [18]. For example, mutations residing in the loops responsible for nucleotide binding (codons 12, 13, and 61) occur with high frequency in the RAS gene family (*KRAS*, *HRAS*, and *NRAS*) [17]; mutations at codons 154, 157, 158, 245, 248, and 273 of *TP53* fall in the DNA binding domain of its protein product [19]; and mutations in PIK3CA form two clusters in the helical (E542K and E545K in exon 9) and catalytic (H1047R in exon 20) domains, respectively [20-23]. In extreme cases, many oncogenes are observed with highly recurrent substitutions that change the same amino acid, such as in the case of the substitution of arginine at codon 132 in isocitrate dehydrogenase 1 (IDH1) protein [24] and the V600 mutation in BRAF [25,26]. Although mutation hotspots have been highly mentioned and investigated in numerous studies, the establishment of a formal definition through quantitative measurements and a systematic exploration of the phenomenon in known cancer genes have not yet been performed. Recently, to measure the clustering magnitude and patterns of mutations, Tamborero *et al*. [12] proposed a straightforward measurement of the magnitude of convergence by counting the number of mutations divided by the distance among mutations. In another work, Reimand and Bader [27] applied a regression model to evaluate the mutation rate around phosphorylation sites, arguing that the rate is higher than that of the whole gene. Notably, nonsense mutations often occur anywhere in the protein-coding sequence and produce truncated proteins [28-30]; thus, nonsense mutations are not usually associated with mutation hotspots.

To this end, we performed a mutation set enrichment analysis (MSEA) to study mutation hotspots in genes and hypothesized that genes with mutation hotspots may serve as candidate cancer genes. Specifically, we introduced two MSEA methods amenable to predicting cancer genes. The first method, MSEA-clust, simulates a walk through the sequences and renders a quantitative measurement of the location and extent to which mutations cluster. MSEA-clust is hypothesis-free, because the convergent regions to be discovered are independent of *a priori* annotations of domains or functional sites. The second method, MSEA-domain, assesses whether a protein domain has a higher mutation rate than in the remaining region of the protein. It requires *a priori* annotations of previously known protein domain structures in each transcript. Accordingly, it is hypothesis-driven. We first demonstrated the power of these two methods using simulated data. Then, we applied them to the Catalog of Somatic Mutations in Cancer (COSMIC) database [31]. In particular, we investigated known cancer genes from the Cancer Gene Census (CGC) [32] collection and found that among the 183 CGC genes that had been detected through SNV/indel analyses in previous studies, approximately 51% can be detected through mutation hotspot analysis, while the remaining approximately 49% of genes do not show a clear pattern of mutation hotspots. The high proportion of genes with mutation hotspots encouraged us to predict additional cancer genes based on mutation clustering patterns. Specifically, we applied these methods to eight cancer types using The Cancer Genome Atlas (TCGA) mutation data for cancer gene prioritization (Table S1 in Additional file 1). We showed that both methods are sensitive to detecting candidate cancer genes, as well as to producing novel discoveries. Through comparison with OncodriveCLUST, an early method aimed at identifying genes whose mutations are biased towards a large spatial clustering, we further showed that our MSEA methods had significantly reduced false discoveries.

## Results

### Power estimation from simulation data

**Figure 1:**
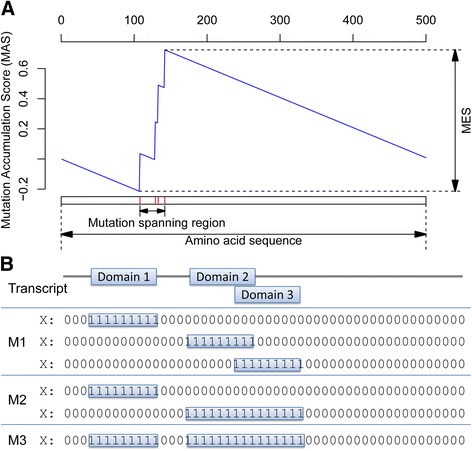
**Illustration of the MSEA methods. (A)** MSEA-clust. The x-axis displays a schematic of the protein amino acid sequence. The red vertical lines indicate mutations affecting the protein sequence, which in turn contribute to the mutation accumulation score (MAS, blue line, y-axis) and mutation enrichment score (MES). **(B)** MSEA-domain. The top portion represents the amino acid sequence of a protein with three domains, two of which overlap. The lower panels display the binomial representation of the M1, M2, and M3 models to interpret protein domains, for which 1 indicates amino acids included in a domain(s), and 0 denotes amino acids not represented by a domain(s).

An overview of MSEA is presented in Figure 1. A detailed description can be found in the Materials and methods section. We estimated the power of the MSEA methods through simulation data in different scenarios. For MSEA-clust, we considered the following factors when generating simulation data: amino acid length, mutation spanning region length, location of mutation spanning region within the gene, the number of mutations, and whether to allow recurrent mutations. The average amino acid length of RefSeq genes is 559, with a median value of 429 and a range between 24 (MTRNR2L1) and 35,991 (TTN). Thus, we selected 500 for protein length in our simulation. The mutation spanning region length was examined at 10, 50, 100, 200, and 300. The location of mutation spanning regions is designed to spread across the protein sequences and ensure that all amino acids in the protein are considered. In practice, we require an eligible protein to have at least four amino acid changes for MSEA analysis. Thus, we simulated mutation data with the number of mutations per transcript as 4 and 8. In each scenario, we generated 100 random datasets and defined the power as the proportion of cases with *p*_clust_ <0.05.

Table S2 in Additional file 1 summarizes the power estimation of the MSEA-clust method. Briefly, the statistical power of MSEA-clust increases when the mutation spanning regions become shorter and the number of mutations becomes larger. Recurrent mutations improve power. On the contrary, power is not influenced by the location of the mutations. These results demonstrate that MSEA-clust has the capability to detect genes with more mutations occurring in converged gene regions, a feature that resembles the observed mutation hotspots in known cancer genes.

For MSEA-domain, we generated simulation data based on the following factors: amino acid length, domain length, domain location in the gene, mutation spanning location, number of mutations, and recurrent mutations. By using domain annotations from the Pfam database [33], the SMART database [34], and the NCBI Conserved Domain Database [35], we found that the median domain length is 92; the average value is 136; and the range is 1 to 3,628. Thus, we selected domain lengths of 100, 200, 300, and 400 in the simulation data. In practice, mutations could occur in certain regions of a domain, for example, phosphorylation sites within a kinase domain. Therefore, we divided each domain into four even regions along the domain (at positions 1 to 25%, 26 to 50%, 51 to 75%, and 76 to 100%), plus an additional scenario for mutations that randomly occur in the whole domain. This design formed five scenarios for each domain. For each scenario, we generated 100 random datasets, and the power was calculated as the proportion of the cases with *p*_domain_ <0.05.

As shown in Table S3 in Additional file 1, the statistical power of MSEA-domain increases with more mutations or smaller domain length but is not influenced by the mutations’ locations within the domains. Surprisingly, power decreased when recurrent mutations were allowed. Several reasons may explain decreased power with recurrent mutations. Recurrent mutations are much more unlikely to occur by themselves by chance and thus, when recurrent mutations are present, the null model h0 tends to be significant already (see Materials and methods). Because the *P*-values in the MSEA-domain model describe whether mutations are significantly associated with the domain distribution (that is, whether the alternative model h1 improves model fitting compared to h0), recurrent mutations did not provide an advantage in this test. These results indicate that MSEA-domain may not be powerful when detecting highly recurrent mutations, for example, the R132H mutation in IDH1.

### Pan-cancer analysis: approximately 51% Mis-CGC genes showed mutation hotspots

**Figure 2:**
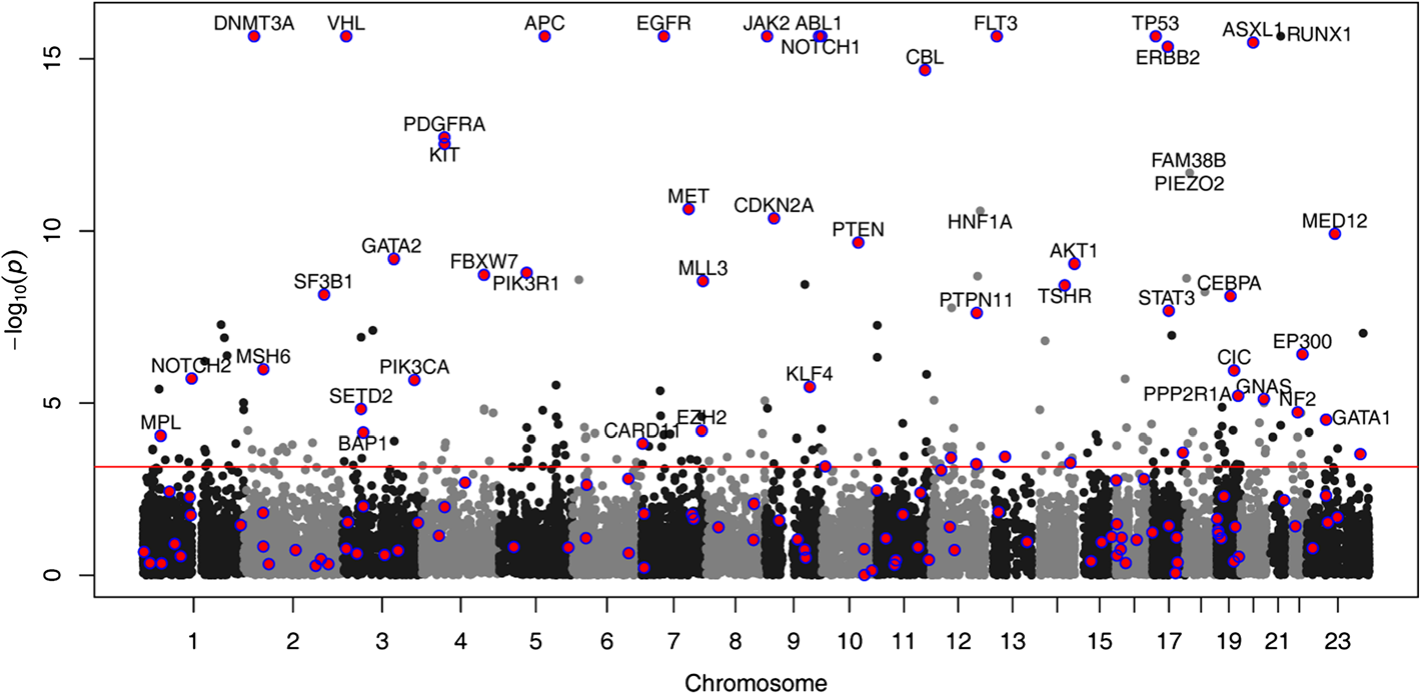
**Manhattan plot of COSMIC genes analyzed by MSEA-domain.** Each dot represents a gene. Purple dots represent Mis-CGC genes (see text). The horizontal red line indicates *P*-value =7 × 10^-4^, at which the FDR is approximately 0.05.

We applied MSEA-clust and MSEA-domain to COSMIC data. Using an adjusted *P*-value <0.05 (Benjamini-Hochberg method), MSEA-clust identified 947 significant genes out of 18,284 eligible genes for analysis; and MSEA-domain found 203 significant genes out of 14,224 eligible genes (Figure 2). As we aim to reveal the feasibility of leveraging mutation hotspots in cancer gene prediction, we specifically explored the status of the 183 Mis-CGC genes (Additional file 2). Here, we refer to Mis-CGC genes as those that have one or more of the following mutation types: missense (SNVs), nonsense (SNVs), splice site (SNVs and indels), or frameshift (indels), and thus, they are eligible for mutation hotspot analysis (see Materials and methods for details). Using MSEA-clust, 170 Mis-CGC genes were eligible for the analysis, that is, they had ≥4 non-silent mutations. Among them, 82 (48.2%) genes had an adjusted *P*-value <0.05 (Benjamini-Hochberg method). Using MSEA-domain, 139 Mis-CGC genes were eligible. As shown in Figure 2, 43 (30.9%) Mis-CGC genes were significant (Benjamini-Hochberg method adjusted *P* <0.05). Collectively, 87 of the 170 Mis-CGC genes (51.2%) were significant (adjusted *P*-values <0.05), indicating that their mutations tended to cluster in certain sequence regions (that is, mutation hotspots). Here, we used Benjamini-Hochberg method adjusted *P*-values <0.05 to define significant genes, compared with the criterion false discovery rate (FDR) <0.2 that was applied in MSEA-domain analysis of the eight cancers. This is because COSMIC data were collected from previously published studies. Due to study biases, the mutation data are highly inflated towards those intensively studied mutations (for example, codons 12, 13, and 61 in *KRAS*). Thus, we used a stringent *P*-value cutoff to define significant genes.

### Overview of results by MSEA-clust and MSEA-domain in eight cancers

We applied MSEA-clust and MSEA-domain in eight TCGA cancers: acute myeloid leukaemia (LAML), breast adenocarcinoma (BRCA), colon and rectal carcinoma (COAD, READ), glioblastoma multiforme (GBM), lung squamous cell carcinoma (LUSC), ovarian serous carcinoma (OvCa), and uterine corpus endometrial carcinoma (UCEC). For each cancer, we obtained six sets of empirical *P*-values using six mutation sets (see Materials and methods), which are 1) all non-silent SNVs, 2) deleterious non-silent SNVs, 3) all non-silent SNVs plus indels, 4) deleterious non-silent SNVs plus indels, 5) all silent (synonymous) SNVs, and 6) all silent SNVs plus benign non-silent SNVs, respectively. Figures S1 to S4 in Additional file 1 show the histograms and Q-Q probability distribution plots of the empirical *P*-values from MSEA-clust, MSEA-domain M1, MSEA-domain M2, and MSEA-domain M3 for each cancer, respectively. The data for LAML were not included in the figure because <50 genes were eligible for LAML analysis and the distributions formed by the small number of genes might not be reliable. Overall, MSEA-clust and MSEA-domain had uniformly distributed *P*-values in most cancer types. Especially for MSEA-domain, the type I error was estimated to be low, according to the Q-Q plot. For MSEA-clust, a slight inflation existed in some cancers (for example, BRCA, GBM, COADREAD, LUSC, and UCEC). Thus, additional filtering is suggested, such as expertise review [36].

To further assess the *P*-value distributions and to identify potential reasons for the slight inflation, we examined several factors that may impact the distributions, for example, non-silent versus silent mutations (Figures S1 to S4 in Additional file 1) and gene expression levels (Figures S5 to S8 in Additional file 1). Silent SNVs do not change amino acid sequences and the results obtained using silent SNVs are expected to reflect the actual situation not related with cancer processes. In our results, the *P*-value distributions obtained using silent SNVs are much closer to the expected distribution. Especially for cancer types where a slight inflation was observed using non-silent mutations by MSEA-clust, for example, GBM, COADREAD, and LUSC, the *P*-value distributions obtained using silent SNVs are close to normal (Figures S1 in Additional file 1). However, in BRCA and UCEC, both non-silent and silent mutation sets showed skewed *P*-value distributions towards 0. This could be partially explained by the recent studies reporting that not only non-silent SNVs but also synonymous SNVs are likely under natural selection in human cancers [37]. Collectively, these results prompted us to take into consideration the silent mutations when evaluating hotspots formed by non-silent mutations, as we did in the step of background adjustment (see Materials and methods).

Gene expression has been previously noted as an important factor associated with mutation rates across the genome [38]. A strong correlation was reported between somatic mutation frequency in cancers and gene expression levels [38]. When applying MSEA-clust on non-silent SNVs and/or indels, we found that expressed genes had a higher proportion of significant genes (those whose nominal *P*-value <0.05) than unexpressed genes in almost all cancer types except LUSC (Figure S5 in Additional file 1, histograms). On the contrary, for silent SNVs and/or benign non-silent SNVs, unexpressed genes had a higher proportion of significant genes in all cancers except OvCa. When using MSEA-domain, the trend is even stronger that expressed genes tend to have a higher proportion of significant genes (Figures S6 to S8 in Additional file 1, Q-Q plot, the dark green points with the sharp departure from the reference line in areas with high -log_10_(*P*) values). These results collectively indicate that gene expression is an important factor that is related to mutation cluster patterns, where non-silent mutations tend to cluster more frequently in expressed genes than in unexpressed genes.

The ratio of non-synonymous SNVs versus synonymous SNVs (*NS/S* ratio, conventionally also called *d*_N_/*d*_S_ or Ka/Ks ratio) is widely used to measure the selection pressure of genes [39]. A higher *NS/S* ratio indicates a positive selection on the corresponding gene. Here, we adapted the concept and defined a ratio using *S/NS*, that is:

$$ S/NS=\frac{\# silent\  SNVs}{\#non- silent\  SNVs} $$

We used this ratio mainly because, for some genes, there are no reported synonymous SNVs, and their *NS/S* ratios would be impossible to measure. Accordingly, a lower *S/NS* ratio indicates a stronger selection pressure on the corresponding gene. We plotted the *S/NS* ratio for genes in different *P*-value intervals obtained by MSEA-clust. As shown in Figure S9 in Additional file 1, the *S/NS* ratio is significantly lower in genes with *P*-values <0.01 obtained by MSEA-clust. This implies that the significant genes identified by MSEA-clust, although a skewed *P*-value distribution is occasionally observed, tend to be under positive selection and are more likely to be driver genes.

#### Genes identified by MSEA-clust using single nucleotide variants

**Figure 3:**
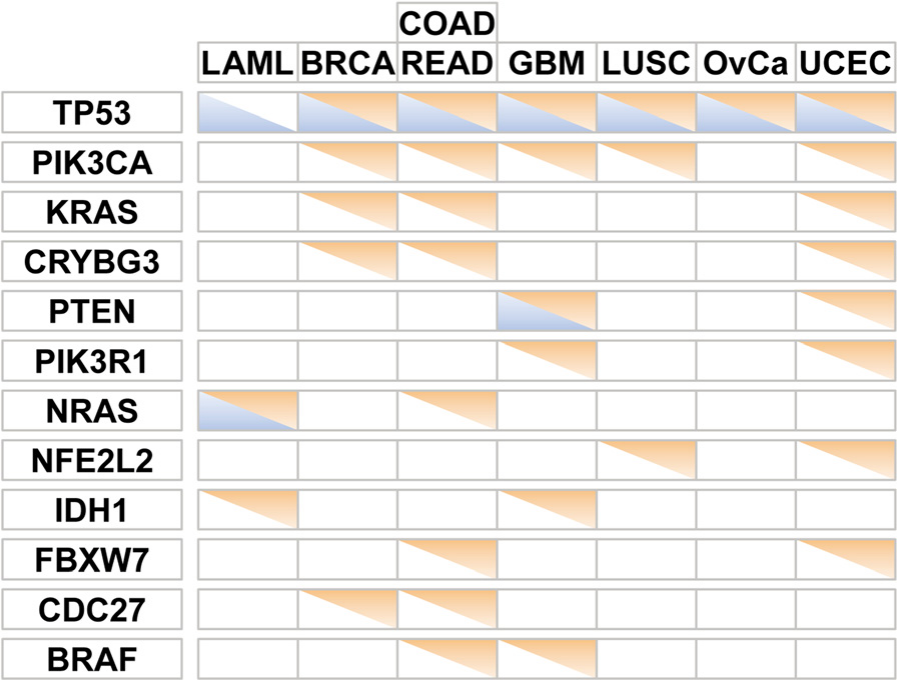
**Significant mutation hotspot genes detected in at least two cancers.** Orange: genes detected by MSEA-clust; blue: genes detected by MSEA-domain. Genes were first ordered by the frequency of their occurrence across cancer types, then alphabetically. Orange or blue triangles represent whether the corresponding gene is detected in the respective cancer type. The size or color of these triangles does not imply the scale of significance.

Using non-silent SNVs as the working mutation data and the silent SNVs as the background data, MSEA-clust identified 63 significant genes whose non-silent SNVs form mutation hotspots in 8 cancers (adjusted *P*-value <0.05). Remarkably, 28 of them were CGC genes as well (44.4%), providing a high proportion of known cancer genes. Here, we used all CGC genes for comparison, instead of using only Mis-CGC genes, in order to incorporate as much *a priori* information as possible on cancer genes. The detailed results of each cancer are shown in Additional file 3. Notably, 12 genes were found in more than one cancer type (Figure 3), and *TP53* was the most frequently observed gene found in all cancers except LAML, followed by *PIK3CA*, *KRAS*, and *CRYBG3*. If using deleterious non-silent SNVs only, MSEA-clust identified 61 significant genes, with 30 (49%) being CGC genes.

The median length of mutation spanning regions is 117 and the average value is 167, indicating that our simulation is appropriate. The minimum region occurred in genes where a mutation recurrently substitutes the same nucleotide or amino acid. For example, the gene *IDH1* has mutations almost exclusively occurring in the 132nd amino acid (c.G395A, p.R132H); seven of eight mutations occurring in U2 small nuclear RNA auxiliary factor 1 (*U2AF1*) in LAML were substitutions at c.C101 (p.S34). Throughout this work, we use the prefix ‘c’ to represent nucleotide changes and the prefix ‘p’ for amino acid changes. The maximum region was observed in the transcripts of adenomatous polyposis coli (*APC*), spanning from 189 to 1,489. By mapping the mutation spanning regions of each gene with known domain regions, we found that the most frequently overlapped domains included 'P53 DNA-binding domain' (domain name: P53; ID: cd08367; 47 occurrences), 'Protein tyrosine kinase' (Pkinase_Tyr; pfam07714; 13 occurrences), 'SH3_Abi1' (Src homology 3 domain of Abl Interactor 1; cd11971; 12 occurrences), 'H_N_K_Ras_like' (Ras GTPase family containing H-Ras, N-Ras and K-Ras4A/4B; cd04138; 8 occurrences), and 'PKc_like' (Protein Kinases, catalytic domain; cl09925; 6 occurrences). Here, a transcript that is found to be significant in one cancer type is counted as one occurrence.

#### Genes identified by MSEA-domain using single nucleotide variants

Using all non-silent SNVs, a total of 32 significant genes (229 transcripts) were identified by MSEA-domain (adjusted *P*-value <0.2,), 15 (46.9%) of which were known cancer genes from the 513 CGC genes. Here we relaxed the *P*-value cutoff to 0.2 so that we could have an adequate number of significant genes for hotspot candidates. All three models could identify significant genes: 80 transcripts by M1, 49 transcripts by M2, and 100 transcripts by M3. This indicates that mutation hotspots are not necessarily restricted to one domain per gene. For ease of description, we represent each gene using its most significant transcript and found that 18 genes were detected with the most significant transcript by M1, one gene by M2 (*PTEN*), and 13 genes by M3. The most frequently enriched gene is *TP53* (all cancers). All of the remaining genes were detected in only one cancer type. The most frequently enriched domain by MSEA-domain was the same by MSEA-clust: 'P53 DNA-binding domain' (domain name: P53; ID: cd08367; 47 occurrences). The other frequently enriched domains were 'S-adenosylmethionine-dependent methyltransferases' (AdoMet_MTases; cd02440; 7 occurrences) and 'Zinc-finger double domain' (zf-H2C2_2; pfam13465; 4 occurrences).

#### MSEA-clust and MSEA-domain provide complementary results using single nucleotide variants

**Figure 4:**
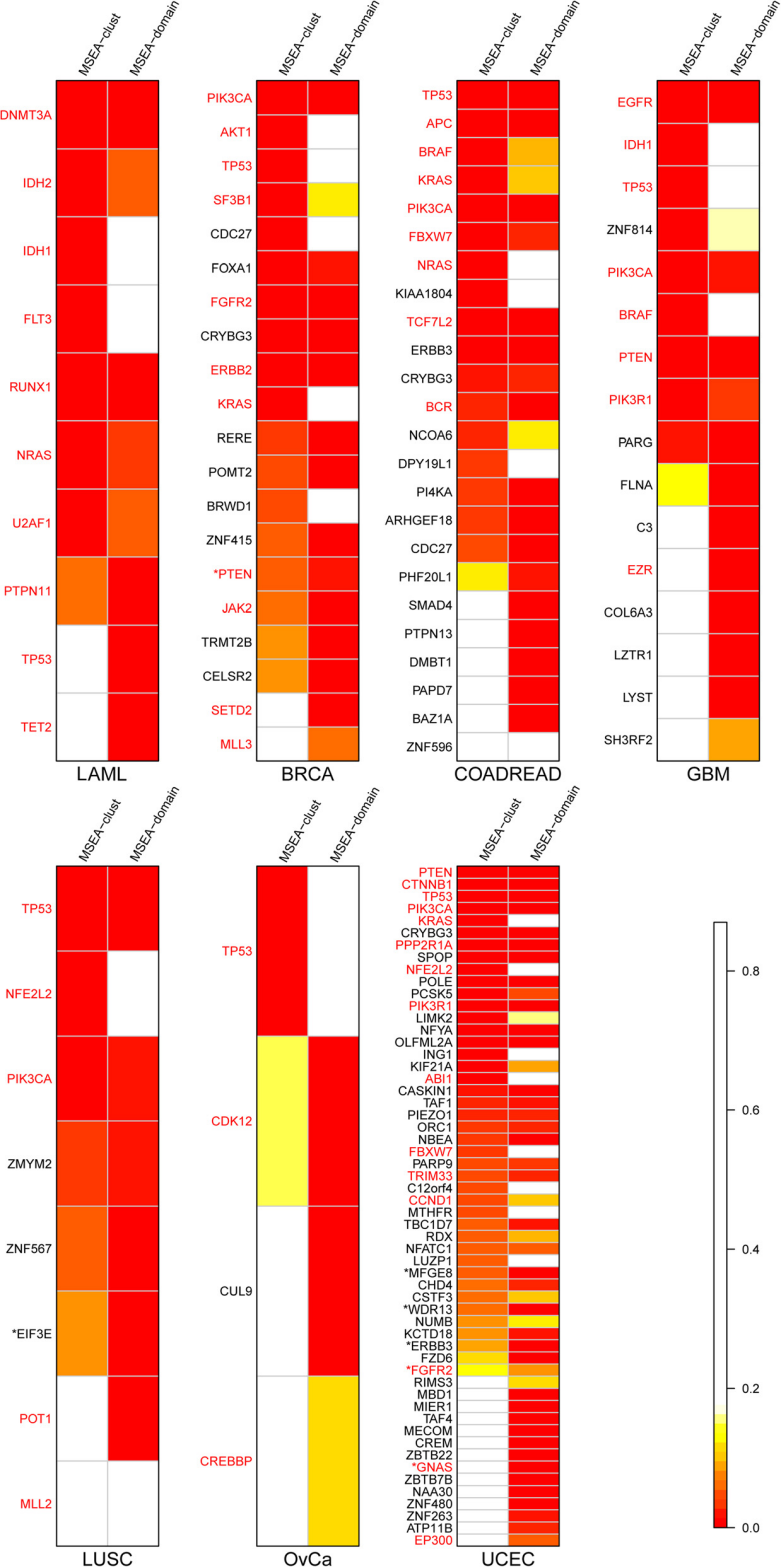
**Significant mutation hotspot genes detected by MSEA-clust or MSEA-domain in each cancer using SNVs only.** Genes in red are those included in the Cancer Gene Census (CGC) collection. Asterisks indicate genes that were only detected when using deleterious non-silent SNVs (see main text). The scale represents *P*-values.

In total, 82 genes were identified by MSEA methods in these cancers when using all SNVs, among which 13 (15.9%) were detected by both methods and 35 (42.7%) were CGC genes (Table 1, Figure 4; Figure S10 in Additional file 1). Among the 82 genes, *TP53* was the most frequently identified gene in terms of cancer types and MSEA methods. The second most popular gene was *PIK3CA*, which was found in six cancers (BRCA, COAD, READ, GBM, LUSC, and UCEC), although it was only identified by the MSEA-clust method (Figure 3).

**Table 1:** **Significant genes identified by MSEA in eight cancers**

	**Number of genes**								
	**LAML**	**BRCA**	**COADREAD**	**GBM**	**LUSC**	**OvCa**	**UCEC**	**Union**	**CGC**
**All non-silent SNVs versus silent SNVs**									
MSEA-clust	7	13	17	9	4	1	33	63	28 (44%)
MSEA-domain	7	7	6	6	2	3	7	32	15 (47%)
Overlap	4	2	2	4	1	1	4	13	8 (62%)
Union	10	18	21	11	5	3	36	82	35 (43%)
CGC (%)	10 (100)	10 (56)	9 (43)	8 (73)	3 (60)	2 (67)	12 (33)		
**Deleterious non-silent SNVs versus silent SNVs**									
MSEA-clust	7	10	13	9	4	2	38	61	30 (49%)
MSEA-domain	7	3	5	9	4	2	17	40	15 (38%)
Overlap	4	1	1	4	1	1	7	13	9 (69%)
Union	10	12	17	14	7	3	48	88	36 (41%)
CGC (%)	10 (100)	9 (75)	9 (53)	7 (50)	4 (57)	3 (100)	14 (29)		
**All non-silent SNVs plus indel versus silent SNVs**									
MSEA-clust	6	106	74	21	8	3	68	251	49 (20%)
MSEA-domain	8	12	10	7	3	4	8	44	16 (36%)
Overlap	3	10	5	4	2	2	6	25	11 (44%)
Union	11	108	79	24	9	5	70	270	54 (20%)
CGC (%)	10 (91)	20 (19)	15 (19)	8 (33)	4 (44)	3 (60)	16 (23)		
**Deleterious non-silent SNVs plus indel versus silent SNVs**									
MSEA-clust	7	81	49	6	2	2	33	162	40 (25%)
MSEA-domain	4	5	5	1	1	1	9	25	9 (36%)
Overlap	2	5	2	1	0	0	2	11	7 (64%)
Union	9	81	52	6	3	3	40	176	42 (24%)
CGC (%)	8 (89)	20 (25)	8 (13)	3 (50)	1 (33)	2 (67)	8 (20)

By examining the genes that were detected by MSEA-clust but not by MSEA-domain, we determined that many had extremely convergent mutation hotspot loci. Examples include the mutations at S34 in U2AF1 [40,41] (LAML), R132 in IDH1 (LAML, GBM), E17K in AKT1 (BRCA), and a four-amino-acid deletion at residues 125 to 128 in TGFBR2 (NM_003242, COADREAD). MSEA-domain failed to detect such mutations, as was consistent with our observations in the simulation data of MSEA-domain’s low power in detecting recurrent mutations. For these genes, the null hypothesis (h0: the mutations are not associated with the domain) was accepted and the alternative hypothesis (h1: the mutations are associated with the domain) was rejected. The high frequency of these mutations is not related to the locations of domain regions; however, it does not exclude the presumption that these mutations indeed converge in a region of the gene. Some other genes are missed because they lack domain annotations, implying that MSEA-clust has a relatively better coverage of eligible genes.

No clear explanatory pattern exists for genes that were detected by MSEA-domain but not by MSEA-clust. However, many have nominally significant *P*-values by MSEA-clust, even though they failed multiple testing corrections, for example, *TP53* in LAML (nominal *p*_clust_ =0.02, *p*_domain_ =5.12 × 10^-3^), *SMAD4* (SMAD family member 4) in COADREAD (nominal *p*_clust_ =0.016, *p*_domain_ =1.43 × 10^-5^), and *CDK12* (cyclin-dependent kinase 12) in OvCa (nominal *p*_clust_ =8.31 × 10^-3^, *p*_domain_ =9.08 × 10^-4^), among others. These results further confirmed that the two methods are complementary; when implemented simultaneously, they could provide better coverage and power.

### Impact of mutation features on mutation hotspots

In addition to using all non-silent SNVs, we also tested mutation hotspots using three sets of mutations: only deleterious SNVs (deleterious missense SNVs and nonsense SNVs), all non-silent SNVs plus indels, and deleterious SNVs plus indels. The null distribution was estimated using all silent SNVs or silent SNVs plus benign missense SNVs, if applicable. For MSEA-clust, six scenarios were created for testing: non-silent SNVs versus the background formed by silent SNVs (NS/S), deleterious non-silent SNVs versus silent SNVs ((del NS)/S), deleterious non-silent SNVs versus silent plus benign missense SNVs ((del NS)/Splus), non-silent SNVs plus indels versus silent SNVs ((NS + I)/S), deleterious non-silent SNVs plus indels versus silent SNVs ((del NS + I)/S), and deleterious non-silent SNVs plus indels versus silent plus benign missense SNVs ((del NS + I)/Splus). For MSEA-domain, four scenarios were created for testing: non-silent SNVs (NS), deleterious non-silent SNVs (del NS), non-silent SNVs plus indels (NS + I), and deleterious non-silent SNVs plus indels (del NS + I). In each of these scenarios, multiple testing corrections were performed independently.

The overall results of each model are summarized in Table 1 and Table S5 and Figures S11 and S12 in Additional file 1. Overall, the significant genes varied among scenarios, but substantial overlap was observed. First, for MSEA-clust, the inclusion of indel data had the most significant impact on the number of significant genes. The inclusion of indels contributed to a noticeable increase of significant genes, especially for BRCA, COADREAD, and UCEC. Second, the selection of the null distribution did not impact the results significantly. When using an estimated null distribution based on either all silent SNVs or silent SNVs plus benign missense SNVs, the significant genes by MSEA-clust did not change substantially.

#### Genes identified using single nucleotide variants plus indels

Genes obtained using both SNVs and indels are presented in Figure S12 in Additional file 1. More significant genes were identified when including indels. Among them, we manually reviewed those non-CGC genes with potential roles in cancer. For example, we found that gene *GIGYF2* (encoding GRB10 interacting GYF protein 2) has a 3-bp deletion (c.3693_3695del, p.1231_1232del) in 11 (6.3%) BRCA samples (Figure S3 in Additional file 1). GIGYF2 may play a role in mediating AKT activity. Previous studies have shown that knockdown of *GIGYF2* resulted in a significant reduction of the phosphorylation of AKT in breast cancer cell lines [42]. Another gene of high interest is *MAP3K4*, which has a 3-bp deletion (c.3566_3568del, p.1189_1190del) occurring in 13 (7.5%) BRCA samples. This deletion is not located in the S_TKc domain; therefore, *MAP3K4* was only detected by MSEA-clust but not by MSEA-domain. *TGFBR2* (transforming growth factor, beta receptor II) was found with a 10-bp frameshift deletion occurring at nucleotides 374 to 383 and changing amino acids 125 to 128 (c.374_383del, p.125_128del).

### Genes with highly convergent mutations

In MSEA-clust, the mutation enrichment score (MES) is determined by the maximum deviation of the mutation accumulation score (MAS), which is calculated by the minimum MAS and the maximum MAS. Accordingly, the region between the locations of the minimum and maximum MAS values pinpoints the most convergent region where mutations would cluster. We specifically examined transcripts whose maximum deviation occurred within three amino acids. In most of these genes, the same or the adjacent amino acids were changed recurrently in many samples. When using SNVs only, most such genes were known cancer genes, such as *U2AF1*, *IDH1*, and *DNMT3A* in LAML (*FLT3* SNVs cluster within five amino acids), *AKT1* and *KRAS* in BRCA, *BRAF* and *KRAS* in COADREAD, *IDH1* and *PARG* (poly (ADP-ribose) glycohydrolase) in GBM, and *KRAS* in UCEC. When using SNVs and indels, more such genes were identified. We plotted the genes whose peak occurs within 3 amino acids and had ≥10 SNVs and indels (Figures S13 to S16 in Additional file 1). Detailed information on these genes, as well as all other significant genes, is provided in Additional files 3 and 4. Due to the limited accuracy of computationally predicted indels from NGS platforms, from which our data were derived, we refrained from highlighting the genes with high confidence. Rather, these genes can be experimentally validated or their function can be explored in the future.

### Informative genes identified by mutation set enrichment analysis

**Figure 5:**
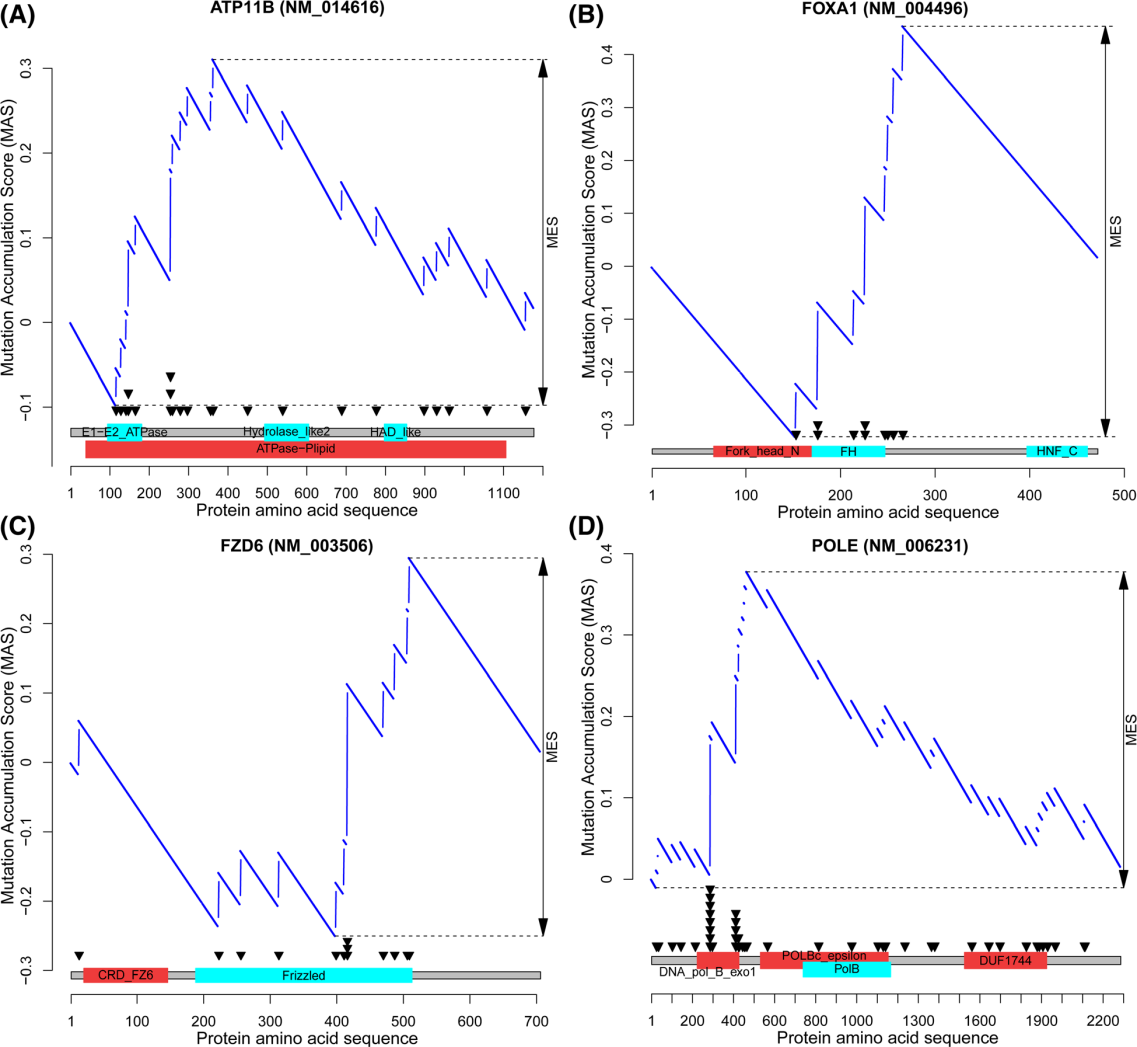
**Example MSEA-clust output for four candidate genes.** In each panel, the top portion shows the MAS score (y-axis) by the MSEA-clust method (see Materials and methods); the bottom part shows the mutation distribution (black triangles) across domains (red/cyan boxes) in the transcript (x-axis). **(A)** *ATP11B* in UCEC. **(B)** *FOXA1* in BRCA. **(C)** *FZD6* in UCEC. **(D)** *POLE* in UCEC.

Beside the known cancer genes in those genes identified by MSEA, there were also novel discoveries, such as new gene-cancer type pairs and novel mutation patterns previously unreported. Some genes had been previously implicated in cancer but were re-found within our results in different cancer types. For example, *ABI1* and *TRIM33* were both known cancer genes and were included in the CGC list, but their mutation patterns in UCEC have been rarely reported, even in the most recent comprehensive studies [16,43] (Figure 5). There are also genes that had been previously studied for their gene expression changes in cancer but whose mutation patterns had not been explored. For example, the altered expression of gene *ATP11B* (encoding ATPase, class VI, type 11B) is associated with cisplatin resistance in ovarian cancer [44]. Here we revealed that its mutations clustered around its E1-E2 ATPase domain (Figure 5). We also found that *FZD6*, a critical gene in the WNT pathway, had mutations clustered in its Frizzled domain in UCEC (Figure 5). In OvCa, although only three genes were significant by MSEA methods, *CUL9*, which encodes an E3 ubiquitin ligase that binds to p53 [45], was identified with mutation clustering in the cullin domain. Put together, our results complemented the previous understanding of cancer genes [16,36,43] by quantitatively pinpointing mutation hotspots, predicting new gene-cancer type pairs, and providing alternative insights.

### Comparison with other tools

To assess the performance of MSEA-clust and MSEA-domain through a comparison to similar tools, we implemented the software OncodriveCLUST [12] on the same simulation data and the same datasets of the eight cancers used for MSEA, respectively. OncodriveCLUST is one of the earliest tools to detect mutation clusters. It assumes unbalanced baseline mutation rates across all gene positions and computes a clustering score based on the proportion of mutations within a cluster and the distances among mutations. During the process of OncodriveCLUST, positions with a number of mutations above a background rate threshold were identified as potential cluster seeds. The threshold was computed according to binomial distribution and gene length. Because of this feature, our simulation data with no recurrent mutations were found ineligible for OncodriveCLUST analysis; otherwise, no meaningful seeds would be identified, that is, all positions failed the background rate. Thus, we could only perform the comparisons using simulation data with recurrent mutations. As shown in Figure S17 in Additional file 1, OncodriveCLUST displayed reduced power in all scenarios compared with MSEA-clust or MSEA-domain. For all simulated genes, if OncodriveCLUST successfully identified a seed position that passed the background mutation rate, it could easily identify the mutation cluster around the position; however, if no such positions were found, OncodriveCLUST failed to detect the hotspot even if four mutations changed four continuous amino acids. On the contrary, MSEA, especially MSEA-clust, could detect mutation hotspots regardless of recurrent mutations.

We applied OncodriveCLUST to the same dataset of eight cancer types with consideration of mutation patterns, as previously used for MSEA; for example, all non-silent SNVs versus silent SNVs and all non-silent SNVs plus indels versus silent SNVs. OncodriveCLUST identified a range of 0 to 323 significant genes (q-value <0.05) per cancer type using SNVs, compared with 1 to 33 by MSEA-clust and 2 to 7 by MSEA-domain. When using SNVs plus indels, the numbers of genes were 19 to 1,014 by OncodriveCLUST, 3 to 106 by MSEA-clust, and 3 to 12 by MSEA-domain. Although there is no gold standard gene list to evaluate both tools, the range of significant genes by OncodriveCLUST seems to be abnormally high. We thus compared the significant genes from OncodriveCLUST and those from MSEA with CGC genes, respectively. As shown in Figures S18 and S19 in Additional file 1, although OncodriveCLUST identified many more genes than MSEA (except in LAML and OvCa), MSEA results showed higher precision in all cancer types than those obtained by OncodriveCLUST. Furthermore, the histogram plots of OncodriveCLUST results implied an inflation of the type I error (Figure S20 in Additional file 1) in all cancer types. These results showed that our MSEA methods performed equally well, if not better, as previously reported methods, and MSEA promises greatly reduced FDRs.

## Discussion

The explosive growth in the number of somatic mutations reported in cancer genomes has placed a high demand on the development of computational tools that can help researchers and clinicians extract and interpret information about the candidate mutations and genes underlying tumorigenesis. In this study, we leveraged the observation of mutation hotspots in known cancer genes to prioritize candidate cancer genes. We proposed two methods based on genes’ mutation clustering patterns, MSEA-clust and MSEA-domain. Through our demonstration of these methods in the COSMIC data and CGC genes, we found that in approximately 51% of Mis-CGC genes, somatic mutations form major clusters that are distinguishable and verify the rationale to prioritize cancer genes based on mutation hotspots. We next applied these methods to data generated from TCGA, encompassing somatic mutations from eight major cancers. Our results highlighted well-established cancer genes and nominated novel candidates. These methods provide valuable tools for future cancer gene studies.

The two methods are complementary to each other. MSEA-clust is more sensitive to detecting genes with highly recurrent mutation hotspot loci (for example, *PIK3CA* and *IDH1*). Such mutations are not detectable by the MSEA-domain method, because the high occurrence is significantly unexpected in random data, regardless of the domain regions. MSEA-clust also has better coverage on proteins that lack domain annotations or proteins in which the substitutions occurred outside of domain regions (for example, *MAP3K4* and *MEF2A* (Myocyte enhancer factor 2A) in BRCA). On the contrary, MSEA-domain is hypothesis-driven and provides a better interpretation of the resultant genes, since most domains are known for their functions. For example, MSEA-domain can be extended for scenarios where regions are defined using biological functional units, such as protein pockets [46], protein secondary structure units [47,48], or regulatory regions (for example, promoters, untranslated regions). In practice, we suggest the user apply both methods and combine the results, in order to find all possible mutation hotspots. However, the user with a specific interest can apply only one of the methods.

The proposed methods have several limitations. First, in the results from MSEA-clust, there appears to be a relatively higher type I error in some cancers, such as COADREAD, GBM, and UCEC (Figure S1 in Additional file 1). The reason for this occurrence remains unknown. Previous studies have shown that replication timing and gene expression are two major reasons for an unbalanced gene-wise mutation rate, for example, genes located in late replication regions have higher mutation rates [49]. We manually examined our results but did not find an overrepresentation of such genes (for example, *CSMD3* and *TTN* [38]) in our list, implying that replication timing may not play an important role in generating 'mutation hotspots' within gene coding regions. This assertion is reasonable, because replication timing typically affects a large genomic region in the chromosome, and it is unlikely that a small region in a gene has different replication timing from other regions in the same gene. Previous works also state that manual filtering is necessary in cluster-based approaches to avoid apparent false discoveries [36]. Even with potential inflations, MSEA-clust nominates candidate genes that may have been missed by the MSEA-domain method, many of which are known CGC genes. Second, not all SNVs and indels used in this work have been experimentally validated, for example, through Sanger re-sequencing. False positive mutations likely exist due to sequencing errors or mapping errors. For example, the 3-bp deletions in *GIGYF2* and *MAP3K4* in BRCA were not validated. Detection of short indels from NGS data tend to have high false positives [50]. In practice, a high quality list of mutations is required to warrant the validity of MSEA results, and caution should be used during follow-up analysis. Nevertheless, the combined list of genes identified by both MSEA methods provides a short list of promising candidate genes. Finally, MSEA methods do not take into consideration the mutation hotspots formed by gene fusion breakpoints or that occurred in regulatory regions (for example, promoter, untranslated region, enhancer, and so on). Such information has not been made publically available, as for SNVs and indels, because most NGS data currently available are based on whole exome sequencing. Since the cost of whole genome sequencing is close to $1000 per genome, numerous whole genome sequencing data will be generated in the near future. Accordingly, MSEA can be extended by considering data such as gene fusion or regulatory information.

Of note, a mutation clustering pattern is one important feature observed in known cancer genes. MSEA detected approximately 51% Mis-CGC genes, while the remaining approximately 49% of genes did not show a clear pattern of mutation hotspots. However, other known features exist in cancer genes. For example, many known cancer genes tend to have an overrepresentation of functionally deleterious mutations [11] or positive selection pressure (for example, a high ratio of non-synonymous versus synonymous SNVs) [39]. Approaches based on these features are also expected to provide more comprehensive candidate gene lists. In addition, the inclusion of other types of somatic mutations, such as somatic copy number variations [51] and translocations, will also nominate candidate cancer genes [38]. Furthermore, tumor suppressor genes are often interrupted by nonsense mutations that could occur anywhere across the gene, and these mutations typically do not form hotspots [20,52]. Thus, MSEA will likely perform poorly in detecting tumor suppressor genes.

Finally, the prioritized candidate genes and their mutations in no way guarantee a 'driver' role of the gene in cancer. Follow-up experimental validation is required to verify the functional impact of the mutations or genes in the corresponding cancer(s). We attempted to interpret the candidate genes for their potential prognostic roles. Indeed, we found several genes with significant prognostic impact on overall survival, such as *NPM1*, *RUNX1*, and *TP53* in LAML, and *PIK3CA*, *TP53*, and *PTEN* in UCEC. Most are well-studied cancer genes. For many novel genes detected by MSEA, they were only mutated with low frequency in the population and did not provide a sufficient number of samples for survival analysis. Thus, future functional analysis is needed to validate and unveil the potential roles of these candidate genes.

## Conclusions

By focusing on somatic SNVs and indels, we proposed two novel methods to detect candidate cancer genes whose somatic mutations tend to form mutation hotspot regions. We explored the prevalence of mutation hotspots in known cancer genes, demonstrated the MSEA approaches in simulated data, and applied them to eight major cancers from TCGA. Our results not only confirmed known cancer genes but also proposed a list of novel candidate genes. Due to the limited data we analyzed, future work is warranted for performance evaluation, method enhancement, and functional validation of novel genes.

## Materials and methods

### Cancer somatic mutation data from COSMIC

We downloaded somatic mutation data from COSMIC (v65, May 20, 2013) [31], a public database that curates somatic mutation information related to human cancers. The downloaded file included 1.15 million lines of data. To avoid redundancy, we defined a mutation (uniquely tagged by a 'mutation.ID' in COSMIC data) in a sample ('Sample.name') as a singular, unique mutation record and kept only one copy of the record. A mutation (for example, T790M in EGFR) could appear in multiple mutation records corresponding to multiple samples. Records with unknown amino acid changes, no genomic positions (GRCh37), or no detectable mRNAs/proteins were excluded. Silent mutations were also excluded. This process resulted in a total of 841,207 non-silent mutation (nonsynonymous SNVs and indels) records for the following analysis.

We downloaded the CGC gene list (December 04, 2013) for known cancer genes [3,53]. The CGC database catalogues genes with causally implicated mutations in cancer. In our work, a total of 513 CGC genes were downloaded. A close inspection of the mutation types involved in these genes showed that more than 60% of genes were found with mutations in the form of amplification, large deletion, or translocation. As we focus on SNVs and indels in this study, we selected those genes that have one or more of the following mutation types: missense (SNVs), nonsense (SNVs), splice site (SNVs and indels), or frameshift (indels). The application of this filtering rule resulted in 183 (35.7%) CGC genes that were eligible for mutation hotspot analysis (Additional file 2). We denoted these genes as Mis-CGC genes, short for 'missense SNV,' which is the most prevalent mutation type in these genes.

### Cancer somatic mutation data from eight TCGA cancers

We retrieved somatic mutation data for eight cancers from TCGA [43]: acute myeloid leukaemia (LAML), breast adenocarcinoma (BRCA), colon and rectal carcinoma (COAD, READ), glioblastoma multiforme (GBM), lung squamous cell carcinoma (LUSC), ovarian serous carcinoma (OvCa), and uterine corpus endometrial carcinoma (UCEC). Colon and rectal carcinomas were merged as one dataset for all the analyses in this study. The original work annotated mutation data using ENSEMBL (version 69) [43]. We updated the functional annotations of mutations using NCBI RefSeq implemented by ANNOVAR [54]. Domains and their locations in each transcript of a gene were downloaded from the NCBI ftp [55]. All ID mapping and conversions were implemented in the R software [56], based on annotation data available at the UCSC Genome Browser [57]. Because different transcripts of the same protein may have different mutation and domain annotations, we took each transcript as a unit, unless otherwise specified.

The somatic mutation data used in our work included somatic SNVs and indels. Throughout this work, we categorized SNVs into silent SNVs and non-silent SNVs. Silent SNVs are synonymous SNVs; non-silent SNVs include missense and nonsense SNVs. For missense SNVs, we used SIFT [9], PolyPhen-2 [4], and MutationAssessor [58] to predict their functional impacts. In particular, if a missense SNV is predicted to be deleterious by any of the three tools, it is defined as a deleterious missense SNV; otherwise, it is considered a benign or tolerable missense SNV. For indels, we considered all as non-silent, regardless of whether they caused frameshift or not. We then created different groups of mutations based on their functional impacts for hotspot detection.

In addition to mutation data, microarray gene expression data for these eight cancer types were obtained from the Cancer Cell Line Encyclopedia [59]. For each cancer, we extracted the cancer tissue-relevant gene expression profile, based on the description of the primary site, histology, and histology subtypes for each cell line [59]. The median gene expression (measured by the normalized robust multi array (RMA) value) across all cancer-relevant cell lines was computed for each gene in each cancer. Genes with an RMA >5 were designated as expressed in the corresponding cancer type [40]. Alternatively, some of the tumors used in our work have matched RNA-sequencing data, as provided by the TCGA data portal. These data can also be used to define expressed genes in future work. Table S1 in Additional file 1 summarizes the data used in this work.

### Mutation set enrichment analysis based on mutation clusters: MSEA-clust

In MSEA-clust, we modified the Kolmogorov-Smirnov test to detect the clustering patterns of mutations along a gene transcript. MSEA-clust simulates a walker walking through the amino acid sequence of a transcript while keeping record of an assessment value, which changes according to the occurrence and frequencies of mutations (Figure 1). The largest variation of the assessment value is indicative of both the location and the magnitude of the clusters formed by mutations. There are four steps in MSEA-clust, as described below.

#### Step 1: calculation of a mutation enrichment score

We define a MAS as the assessment value. MAS is a vector of length *L*, where *L* is the amino acid length of the transcript. Starting with 0, MAS is recorded at each position while we walk down the sequence of the transcript, and the *i*^th^ element of MAS is the assessment value for the *i*^th^ position. MAS increases when we encounter a mutation and decreases at non-mutated positions. As aforementioned, a mutation record refers to a mutation in a sample; thus, at the *i*^th^ position, there could be multiple mutation records, referring to multiple samples in which the mutation occurs. We define a vector *Y* of length *L* to indicate the number of mutation records at each position, that is, *Y* = (*y*_1_, …, *y*_*L*_). Specifically, for locations where no mutation occurs, *y*_*j*_ =0. We also define a vector *L*^M^ to record the positions where any mutation occurs. The magnitude of increment and decrement of MAS is computed by *S*_*inc*_ =1/(Number of mutation records) =1/∑*Y*, and *S*_*dec*_ =1/(Number of non‐mutated positions), respectively. Here, the sum of increment equals the sum of decrement, both of which are 1. The increment at a mutated position is calculated by *y*_*j*_ × *S*_*inc*_, where 1 ≤ *j* ≤ *L* and *j* ∈ *L*^*M*^. The decrease at all non-mutated positions is always *S*_*dec*_. Thus, MAS at the *i*^th^ position is calculated by:

$$ MA{S}_i={\displaystyle \sum_{j\in {L}^M,\ j\le i}{y}_j\times {S}_{inc}}-{\displaystyle \sum_{j\notin {L}^M,j\le i}{S}_{dec}} $$

where 1 ≤ *i* ≤ *L*. Based on the definitions of increment and decrement, we have MAS_*L*_ =0. Therefore, MAS bridges between 0’s at the starting and ending positions, with an expectation of a sharp increment within a short distance in the sequence regions where many mutations cluster. Accordingly, the maximum departure observed in the recorded value MAS will indicate where mutations cluster. We thus define the MES as the maximum deviation of MAS across the transcript (Figure 1):

$$ MES= \max (MAS)- \min (MAS) $$

#### Step 2: statistical significance test

We estimate the significance of MES using a randomization-based test. Given a transcript, we randomly select the same number of true mutation records across the amino acid sequence of the transcript in each randomization process, allowing for replacement. The rationale behind the allowance of replacement is to incorporate recurrent mutations. For the randomly selected mutations, a MES is computed following step 1 and denoted as MES(π). The randomization process is implemented a total number of 10 × *L* times to ensure a sufficient shuffle. The resultant MES(π) values form a null distribution of MES for a given transcript expected at random. Based on this method, a normalized MES, denoted as NES, as well as an empirical *P-value*, can be computed by:

$$ NES=\frac{MES- mean\left(MES\left(\pi \right)\right)}{sd\left(MES\left(\pi \right)\right)}\ \mathrm{and}\ p=\frac{\#\left\{MES\left(\pi \right)\ge MES\right\}}{1+10\times L} $$

In this way, the NES for each transcript is independent of its number of mutations or its amino acid length, and thus, different transcripts are comparable to each other. Depending on the intended purposes, the NES value is mainly used for significance estimation, while the empirical *P-value*s are mainly used for the measurement of overall inflation.

#### Step 3: background adjustment and null distribution estimation

We propose two strategies of background adjustment. The first strategy is a unit-based mutation hotspot adjustment. For each transcript unit, we test for its potential mutation hotspots using 1) non-silent mutations and 2) silent mutations. If a transcript’s silent mutations form any mutation hotspots at nominal significance (nominal *P-value* <0.05), the hotspots formed by non-silent mutations of this transcript will not be considered significant.

Second, we estimate the null distribution of NES using two background SNV sets. The first background set includes all silent SNVs. In contrast, missense SNVs, nonsense SNVs, and indels are considered non-silent mutations and are used for hotspot detection. The second background set includes all silent SNVs plus benign missense SNVs (see 'Cancer somatic mutation data from eight TCGA cancers' section). Accordingly, deleterious missense SNVs, nonsense SNVs, and indels are considered non-silent mutations for hotspot detection.

To accurately estimate the null distribution of NES, we pool the NES obtained by background mutations for all genes. The empirical null distribution is a normal distribution with empirically estimated mean and standard deviation. We used the *locfdr* package in R to obtain the estimation. Specifically, the following steps are implemented: 1) NES values obtained using background mutations are median-centered and used for the estimation of the mean and standard deviation of the null distribution f0 using maximum likelihood iteration; 2) NES values obtained using non-silent mutations, which are also median-centered, are then adjusted using the mean and standard deviation of f0 from step 1, resulting in normalized *z*-scores; and 3) nominal *P-value*s are computed based on the normalized *z* -scores.

#### Step 4: adjustment for multiple testing

We use the Benjamini-Hochberg method [60] to control for a FDR. Genes with an adjusted *P-value* <0.05 are considered significant, unless otherwise specified.

### Mutation set enrichment analysis based on domain mutation rate: MSEA-domain

MSEA-domain extends a generalized linear regression model to examine whether the incidence of mutations is in association with a pre-defined domain. Assuming mutations occur in a gene with a background mutation rate μ, the incidence of mutation events is generally assumed to follow a Poisson distribution:

$$ P\left(y;\mu \right)=\frac{\mu^y \exp \left(-\mu \right)}{y!} $$

where *y* is the number of mutations. When a domain has a higher mutation rate than the remaining regions of the gene, more mutations will be observed in the domain than in other regions, and it is expected that the unbalanced mutation will be detectable. Based on this hypothesis, we developed MSEA-domain to test whether a domain(s) has an abnormal mutation rate by formulating a regression model. In this model, the dependency between mutation events and the predictors is expressed as *E*(*Y*|*X*) = exp(*β*_0_ + *β*_1_*X*), or ln(*E*(*Y*|*X*)) = *β*_0_ + *β*_1_*X*, where *Y* is a vector representing the number of observed mutations at each position along the transcript, and *X* is the domain indicator. Both *Y* and *X* have a length of *L*; here *L* is the amino acid length of the transcript. In the vector *X*, only values 0 and 1 are allowed, where 0 indicates that a position does not belong to a domain and 1 indicates that the position is included in the domain. Considering that the domain structure of a protein can be very complex - for example, multiple domains may co-exist and overlap with each other in a transcript - we designed three models for *X* (Figure 1). In model 1 (M1), each domain is tested independently, and a transcript has multiple *P-value*s, one for each domain. In model 2 (M2), overlapping domains are merged and denoted as a single 'domain region'. Each domain region is then examined, and a transcript has multiple *P-value*s, one for each domain region. In model 3 (M3), all domains are denoted as regions of interest, and each transcript is tested only once, resulting in one *P-value*.

To test whether mutations are associated with the domains in a transcript, we designed the null model as h0: *E*(*Y*|*X*) = exp(*β*_0_), and the alternative model as: h1: *E*(*Y*|*X*) = exp(*β*_0_ + *β*_1_*X*). In practice, due to the sparseness of mutation data, many positions in a transcript do not harbor any mutations, and the data are zero-inflated. Therefore, we utilized the negative-binomial distribution to replace the Poisson distribution. A log-likelihood ratio test was employed to compare the null model h0 and the alternative model h1. For each transcript, the lowest *P-value* among tests of M1, M2, and M3 was selected to represent the transcript level *P-value*.

### Software availability

The code and data used in this work are available at GitHub [61] and our website [62].

## Additional files

Additional file 1: Table S1. Sample description. **Table S2.** Power estimation for MSEA-clust. **Table S3.** Power estimation for MSEA-domain. **Table S5.** Summary of results by MSEA-clust in eight cancer types. **Figures S1-S4.** Histograms and Q-Q plot of *p*-values obtained by MSEA-clust (Figure S1), MSEA-domain (M1) (Figure S2), MSEA-domain (M2) (Figure S3), and MSEA-domain (M3) (Figure S4) for each cancer using different mutations. **Figures S5-S8.** Histograms and Q-Q plot of *p-*values obtained by MSEA-clust (Figure S5), MSEA-domain (M1) (Figure S6), MSEA-domain (M2) (Figure S7), and MSEA-domain (M3) (Figure S8) for each cancer using different mutations in expressed (dark green) and unexpressed (light green) genes. **Figure S9.** The ratio of silent SNVs vs. non-silent SNVs in each cancer. **Figure S10.** Results comparison between different models. **Figure S11.** Comparison of significant genes by MSEA-clust and MSEA-domain in each cancer using SNVs only. **Figure S12.** Comparison of significant genes by MSEA-clust and MSEA-domain in each cancer using SNVs and indels. **Figures S13-S16.** Genes of interest (peak within 3 amino acids) in BRCA (Figure S13), COADREAD (Figure S14), GBM (Figure S15), and UCEC (Figure S16) that were uniquely detected when including indels. **Figure S17.** Power estimation of MSEA and OncodriveCLUST. **Figure S18.** Venn diagram of significant genes identified by MSEA and OncodriveCLUST in comparison with CGC genes. **Figure S19.** Venn diagram of significant genes identified by MSEA and OncodriveCLUST in comparison with CGC genes. **Figure S20.** Histograms of gene-based *p-*values by OncodriveCLUST for each cancer. Additional file 2: Table S4. Detailed information for 183 Mis-CGC genes. Additional file 3: Table S6. Significant genes identified by MSEA using SNVs. Additional file 4: Table S7. Significant genes identified by MSEA using SNVs and indels.

## Abbreviations

BRCA: breast adenocarcinoma
CGC: Cancer Gene Census
COAD: colon carcinoma
COADREAD: colon and rectal carcinoma
COSMIC: Catalog of Somatic Mutations in Cancer
FDR: false discovery rate
GBM: glioblastoma multiforme
LAML: acute myeloid leukemia
LUSC: lung squamous cell carcinoma
MAS: mutation accumulation score
MES: mutation enrichment score
MSEA: mutation set enrichment analysis
NES: normalized mutation enrichment score
NGS: next-generation sequencing
OvCa: ovarian serous carcinoma
READ: rectal carcinoma
RMA: robust multi array
SNV: single nucleotide variant
TCGA: The Cancer Genome Atlas
UCEC: uterine corpus endometrial carcinoma
