# Supplementary material for: MSEA: detection and quantification of mutation hotspots through mutation set enrichment analysis
Source: Genome Biol. 2014 Oct 28;15(10):489. doi: 10.1186/s13059-014-0489-9 (PMC4226881; doi:10.1186/s13059-014-0489-9)
Supplement: Additional file 1: Table S1. — Sample description. Table S2. Power estimation for MSEA-clust. Table S3. Power estimation for MSEA-domain. Table S5. Summary of results by MSEA-clust in eight cancer types. Figures S1-S4. Histograms and Q-Q plot of p-values obtained by MSEA-clust (Figure S1), MSEA-domain (M1) (Figure S2), MSEA-domain (M2) (Figure S3), and MSEA-domain (M3) (Figure S4) for each cancer using different mutations. Figures S5-S8. Histograms and Q-Q plot of p-values obtained by MSEA-clust (Figure S5), MSEA-domain (M1) (Figure S6), MSEA-domain (M2) (Figure S7), and MSEA-domain (M3) (Figure S8) for each cancer using different mutations in expressed (dark green) and unexpressed (light green) genes. Figure S9. The ratio of silent SNVs vs. non-silent SNVs in each cancer. Figure S10. Results comparison between different models. Figure S11. Comparison of significant genes by MSEA-clust and MSEA-domain in each cancer using SNVs only. Figure S12. Comparison of significant genes by MSEA-clust and MSEA-domain in each cancer using SNVs and indels. Figures S13-S16. Genes of interest (peak within 3 amino acids) in BRCA (Figure S13), COADREAD (Figure S14), GBM (Figure S15), and UCEC (Figure S16) that were uniquely detected when including indels. Figure S17. Power estimation of MSEA and OncodriveCLUST. Figure S18. Venn diagram of significant genes identified by MSEA and OncodriveCLUST in comparison with CGC genes. Figure S19. Venn diagram of significant genes identified by MSEA and OncodriveCLUST in comparison with CGC genes. Figure S20. Histograms of gene-based p-values by OncodriveCLUST for each cancer. [file 13059_2014_489_MOESM1_ESM.docx]

**Supplementary material for “Detection and quantification of mutation hotspots through mutation set enrichment analysis (MSEA)”**

Peilin Jia^1,2^, Quan Wang^1^, Qingxia Chen^1,3^, Katherine E. Hutchinson^4^, William Pao^4,5^, and Zhongming Zhao^1,2,4,6,*^

Table S1. Sample description

Table S2. Power estimation for MSEA-clust. All simulation data was based on a transcript length of 500.

Table S3. Power estimation for MSEA-domain. All simulation data was based on a transcript length of 500.

Table S4. Detailed information for 183 Mis-CGC genes.

Table S5. Summary of results by MSEA-clust in eight cancer types.

Figure S1. Histograms and Q-Q plot of *p*-values obtained by MSEA-clust for each cancer using different mutations. For each cancer, *p-*values obtained using six mutation sets were plotted: all non-silent SNVs, deleterious non-silent SNVs, all non-silent SNVs plus indels, deleterious non-silent SNVs plus indels, all silent (synonymous) SNVs, and all silent SNVs plus benign non-silent SNVs. In the histograms, x-axis denotes *p-*value and y-axis denotes the proportion of genes in each *p-*value interval. In the Q-Q plot, the x-axis is the expected *p*-value and the y-axis is the observed *p*-value. The red line indicates the reference line y=x.

Figure S2. Q-Q plot of *p-*values obtained by MSEA-domain (M1) for each cancer using different mutations (see legend of Figure S1).

Figure S3. Q-Q plot of *p-*values obtained by MSEA-domain (M2) for each cancer using different mutations.

Figure S4. Q-Q plot of *p-*values obtained by MSEA-domain (M3) for each cancer using different mutations.

Figure S5. Q-Q plot of *p-*values obtained by MSEA-clust for each cancer using different mutations in expressed (dark green) and unexpressed (light green) genes.

Figure S6. Q-Q plot of *p-*values obtained by MSEA-domain (M1) for each cancer using different mutations in expressed (dark green) and unexpressed (light green) genes.

Figure S7. Q-Q plot of *p-*values obtained by MSEA-domain (M2) for each cancer using different mutations in expressed (dark green) and unexpressed (light green) genes.

Figure S8. Q-Q plot of *p-*values obtained by MSEA-domain (M3) for each cancer using different mutations in expressed (dark green) and unexpressed (light green) genes.

Figure S9. The ratio of silent SNVs vs. non-silent SNVs in each cancer. x-axis: *p*-value. y-axis: the *S/NS* ratio, i.e., $S/NS=\frac{\#silent SNVs}{\#non-silent SNVs}$. Del: deleterious.

Figure S10. Results comparison between different models. (A) MSEA-clust results in each cancer type. Top panel presents comparion among genes obtained using SNVs only in each of the following scenario: (1) non-silent SNVs vs. the background formed by silent SNVs (NS/S), (2) deleterious non-silent SNVs vs. silent SNVs (del NS/S), (3) deleterious non-silent SNVs vs. silent plus benign missense SNVs (del NS/Splus). Bottom panel presents comparison among genes obtained using SNVs plus indels in each of the following scenarios: (4) non-silent SNVs plus indels vs. silent SNVs (NS+I/S), (5) deleterious non-silent SNVs plus indels vs. silent SNVs (del NS+I/S), and (6) deleterious non-silent SNVs plus indels vs. silent plus benign missense SNVs (del NS+I/Splus). (B) MSEA-domain results in each cancer type. The genes obtained in each of the four scenarios were compared: (1) non-silent SNVs (NS), (2) deleterious non-silent SNVs (del NS), (3) non-silent SNVs plus indels (NS+I), and (4) deleterious non-silent SNVs plus indels (del NS+I).

Figure S11. Comparison of significant genes by MSEA-clust and MSEA-domain in each cancer using SNVs only. Genes in grey were only detected when using all non-silent SNVs. Genes in black were detected both when using all non-silent SNVs and when using deleterious non-silent SNVs.

Figure S12. Comparison of significant genes by MSEA-clust and MSEA-domain in each cancer using SNVs and indels. Genes in grey were only detected when using all non-silent SNVs. Genes in black were detected both when using all non-silent SNVs and when using deleterious non-silent SNVs.

Figure S13. Genes of interest in BRCA that were uniquely detected when including indels. Pink triangle indicates indels. Due to space limitation, we only draw genes with ≥10 mutations (SNVs and indels).

Figure S14. Genes of interest (peak within 3 amino acids) in COADREAD that were uniquely detected when including indels. Pink triangle indicates indels. Due to space limitation, we only draw genes with ≥10 mutations (SNVs and indels) and with domain annotations.

Figure S15. Genes of interest (peak within 3 amino acids) in GBM that were uniquely detected when including indels. Pink triangle indicates indels. Due to space limitation, we only draw genes with ≥10 mutations (SNVs and indels) and with domain annotations.

Figure S16. Genes of interest (peak within 3 amino acids) in UCEC that were uniquely detected when including indels. Pink triangle indicates indels. Due to space limitation, we only draw genes with ≥10 mutations (SNVs and indels) and with domain annotations.

Figure S17. Power estimation of MSEA and OncodriveCLUST in different scenarios. Top panels: MSEA-clust vs. OncodriveCLUST; bottom panels: MSEA-domain vs. OncodriveCLUST. Red box: MSEA; blue box: OncodriveCLUST (ODC). In the x-axis, s denotes spanning regions (top panels), and L denotes domain location (bottom panel). All results are based on the same simulation data as used in Table S2 with recurrent mutations allowed.

Figure S18. Venn diagram of significant genes identified by MSEA and OncodriveCLUST in comparison with CGC genes. These results were obtained using all non-silent SNVs. Red bar: MSEA; blue bar: OncodriveCLUST.

Figure S19. Venn diagram of significant genes identified by MSEA and OncodriveCLUST in comparison with CGC genes. These results were obtained using all non-silent SNVs plus indels. Red bar: MSEA; blue bar: OncodriveCLUST.

Figure S20. Histograms of gene-based *p-*values by OncodriveCLUST for each cancer.

Table S1. Sample description

| **Cancer** | **ID** | **# of samples** | **CCLE name** | **# of cell lines** |
| --- | --- | --- | --- | --- |
| Acute myeloid leukaemia | LAML | 195 | AML | 34 |
| Breast adenocarcinoma | BRCA | 771 | Breast | 56 |
| Colon and rectal carcinoma | COAD, READ | 224 | colorectal | 56 |
| Glioblastoma multiforme | GBM | 291 | glioma | 46 |
| Lung squamous cell carcinoma | LUSC | 177 | lung_NSC squamous cell carcinoma | 23 |
| Ovarian serous carcinoma | OvCa | 316 | ovary | 47 |
| Uterine corpus endometrial carcinoma | UCEC | 248 | endometrium | 28 |

Table S2. Power estimation for MSEA-clust. All simulation data was based on a transcript length of 500.

| **Span = 10** | |  | **Span = 50** | |  | **Span = 100** | |  | **Span = 200** | |  | **Span = 300** | |
| --- | --- | --- | --- | --- | --- | --- | --- | --- | --- | --- | --- | --- | --- |
| **Location** | **Power (%)** |  | **Location** | **Power (%)** |  | **Location** | **Power (%)** |  | **Location** | **Power (%)** |  | **Location** | **Power (%)** |
| *# mutations = 4, recurrent = F* | | | | | | | | | | | | | |
| 1 − 10 | 100 |  | 1 − 50 | 100 |  | 1 − 100 | 100 |  | 1 − 200 | 46 |  | 1 − 300 | 16 |
| 101 − 110 | 100 |  | 101 − 150 | 100 |  | 101 − 200 | 100 |  | 51 − 250 | 39 |  | 51 − 350 | 17 |
| 201 − 210 | 100 |  | 201 − 250 | 100 |  | 201 − 300 | 100 |  | 101 − 300 | 45 |  | 101 − 400 | 11 |
| 301 − 310 | 100 |  | 301 − 350 | 100 |  | 301 − 400 | 100 |  | 151 − 350 | 49 |  | 151 − 451 | 15 |
| 401 − 410 | 100 |  | 401 − 450 | 100 |  | 401 − 500 | 100 |  | 201 − 400 | 36 |  | 201 − 500 | 22 |
| *# mutations = 4, recurrent = T* | | | | | | | | | | | | | |
| 1 − 10 | 100 |  | 1 − 50 | 100 |  | 1 − 100 | 100 |  | 1 − 200 | 41 |  | 1 − 300 | 14 |
| 101 − 110 | 100 |  | 101 − 150 | 100 |  | 101 − 200 | 100 |  | 51 − 250 | 48 |  | 51 − 350 | 9 |
| 201 − 210 | 100 |  | 201 − 250 | 100 |  | 201 − 300 | 100 |  | 101 − 300 | 40 |  | 101 − 400 | 20 |
| 301 − 310 | 100 |  | 301 − 350 | 100 |  | 301 − 400 | 100 |  | 151 − 350 | 44 |  | 151 − 451 | 16 |
| 401 − 410 | 100 |  | 401 − 450 | 100 |  | 401 − 500 | 100 |  | 201 − 400 | 45 |  | 201 − 500 | 19 |
| *# mutations = 8, recurrent = F* | | | | | | | | | | | | | |
| 1 − 10 | 100 |  | 1 − 50 | 100 |  | 1 − 100 | 100 |  | 1 − 200 | 69 |  | 1 − 300 | 21 |
| 101 − 110 | 100 |  | 101 − 150 | 100 |  | 101 − 200 | 100 |  | 51 − 250 | 58 |  | 51 − 350 | 27 |
| 201 − 210 | 100 |  | 201 − 250 | 100 |  | 201 − 300 | 100 |  | 101 − 300 | 65 |  | 101 − 400 | 25 |
| 301 − 310 | 100 |  | 301 − 350 | 100 |  | 301 − 400 | 100 |  | 151 − 350 | 61 |  | 151 − 451 | 18 |
| 401 − 410 | 100 |  | 401 − 450 | 100 |  | 401 − 500 | 100 |  | 201 − 400 | 61 |  | 201 − 500 | 20 |
| *# mutations = 8, recurrent = T* | | | | | | | | | | | | | |
| 1 − 10 | 100 |  | 1 − 50 | 100 |  | 1 − 100 | 100 |  | 1 − 200 | 100 |  | 1 − 300 | 45 |
| 101 − 110 | 100 |  | 101 − 150 | 100 |  | 101 − 200 | 100 |  | 51 − 250 | 100 |  | 51 − 350 | 39 |
| 201 − 210 | 100 |  | 201 − 250 | 100 |  | 201 − 300 | 100 |  | 101 − 300 | 100 |  | 101 − 400 | 47 |
| 301 − 310 | 100 |  | 301 − 350 | 100 |  | 301 − 400 | 100 |  | 151 − 350 | 100 |  | 151 − 451 | 41 |
| 401 − 410 | 100 |  | 401 − 450 | 100 |  | 401 − 500 | 100 |  | 201 − 400 | 100 |  | 201 − 500 | 36 |

Table S3. Power estimation for MSEA-domain. All simulation data was based on a transcript length of 500.

| **Domain location** | **Span = 1-25%** | **Span = 26-50%** | **Span = 51-75%** | **Span = 76-100%** | **Span = 1-500** |
| --- | --- | --- | --- | --- | --- |
|  | **Power (%)** | **Power (%)** | **Power (%)** | **Power (%)** | **Power (%)** |
| Domain = 100 | | | | | |
| *# mutations = 4, recurrent =F* | | | | | |
| 1 − 100 | 100 | 100 | 100 | 100 | 100 |
| 101 − 200 | 100 | 100 | 100 | 100 | 100 |
| 201 − 300 | 100 | 100 | 100 | 100 | 100 |
| 301 − 400 | 100 | 100 | 100 | 100 | 100 |
| 401 − 500 | 100 | 100 | 100 | 100 | 100 |
| *# mutations = 4, recurrent =T* | | | | | |
| 1 − 100 | 99 | 100 | 97 | 100 | 100 |
| 101 − 200 | 99 | 100 | 100 | 99 | 100 |
| 201 − 300 | 99 | 99 | 100 | 98 | 100 |
| 301 − 400 | 98 | 100 | 99 | 100 | 100 |
| 401 − 500 | 99 | 100 | 100 | 100 | 100 |
| *# mutations = 8, recurrent =F* | | | | | |
| 1 − 100 | 100 | 100 | 100 | 100 | 100 |
| 101 − 200 | 100 | 100 | 100 | 100 | 100 |
| 201 − 300 | 100 | 100 | 100 | 100 | 100 |
| 301 − 400 | 100 | 100 | 100 | 100 | 100 |
| 401 − 500 | 100 | 100 | 100 | 100 | 100 |
| *# mutations = 8, recurrent =T* | | | | | |
| 1 − 100 | 100 | 99 | 100 | 99 | 100 |
| 101 − 200 | 99 | 100 | 96 | 99 | 100 |
| 201 − 300 | 99 | 100 | 99 | 96 | 100 |
| 301 − 400 | 99 | 100 | 100 | 100 | 100 |
| 401 − 500 | 100 | 100 | 100 | 100 | 100 |
| Domain = 300 |  |  |  |  |  |
| *# mutations = 4, recurrent =F* | | | | | |
| 1 − 300 | 100 | 100 | 100 | 100 | 100 |
| 101 − 400 | 100 | 100 | 100 | 100 | 100 |
| 201 − 500 | 100 | 100 | 100 | 100 | 100 |
| *# mutations = 4, recurrent =T* | | | | | |
| 1 − 300 | 95 | 94 | 86 | 92 | 98 |
| 101 − 400 | 93 | 95 | 88 | 94 | 98 |
| 201 − 500 | 94 | 93 | 95 | 93 | 100 |
| *# mutations = 8, recurrent =F* | | | | | |
| 1 − 300 | 100 | 100 | 100 | 100 | 100 |
| 101 − 400 | 100 | 100 | 100 | 100 | 100 |
| 201 − 500 | 100 | 100 | 100 | 100 | 100 |
| *# mutations = 8, recurrent =T* | | | | | |
| 1 − 300 | 100 | 100 | 100 | 100 | 100 |
| 101 − 400 | 100 | 100 | 100 | 100 | 100 |
| 201 − 500 | 100 | 100 | 100 | 100 | 100 |

Table S4. Detailed information for 183 Mis-CGC genes (see xlsx file).

Table S5. Summary of results by MSEA-clust in eight cancer types.

|  | LAML | | BRCA | | COADREAD | | GBM | | LUSC | | OvCa | | UCEC | |
| --- | --- | --- | --- | --- | --- | --- | --- | --- | --- | --- | --- | --- | --- | --- |
|  | #t | #g | #t | #g | #t | #g | #t | #g | #t | #g | #t | #g | #t | #g |
| All non-silent SNVs vs. silent SNVs | 15 | 7 | 43 | 13 | 37 | 17 | 27 | 9 | 21 | 4 | 15 | 1 | 82 | 33 |
| Del non-silent SNVs vs. silent SNVs | 15 | 7 | 39 | 10 | 34 | 13 | 27 | 9 | 19 | 4 | 17 | 2 | 88 | 38 |
| Del non-silent SNVs vs. silent SNVs & benign missense SNVs | 15 | 7 | 38 | 9 | 34 | 13 | 27 | 9 | 17 | 3 | 17 | 2 | 80 | 32 |
| All non-silent SNVs & indels vs. silent SNVs | 15 | 6 | 204 | 106 | 153 | 74 | 49 | 21 | 22 | 8 | 18 | 3 | 144 | 68 |
| Del non-silent SNVs & indels vs. silent SNVs | 17 | 7 | 159 | 81 | 91 | 49 | 14 | 6 | 3 | 2 | 6 | 2 | 56 | 33 |
| Del non-silent SNVs & indels vs. silent SNVs & benign missense SNVs | 17 | 7 | 154 | 76 | 80 | 40 | 13 | 6 | 2 | 1 | 6 | 2 | 44 | 25 |

Del: deleterious; t: transcript; g: gene.


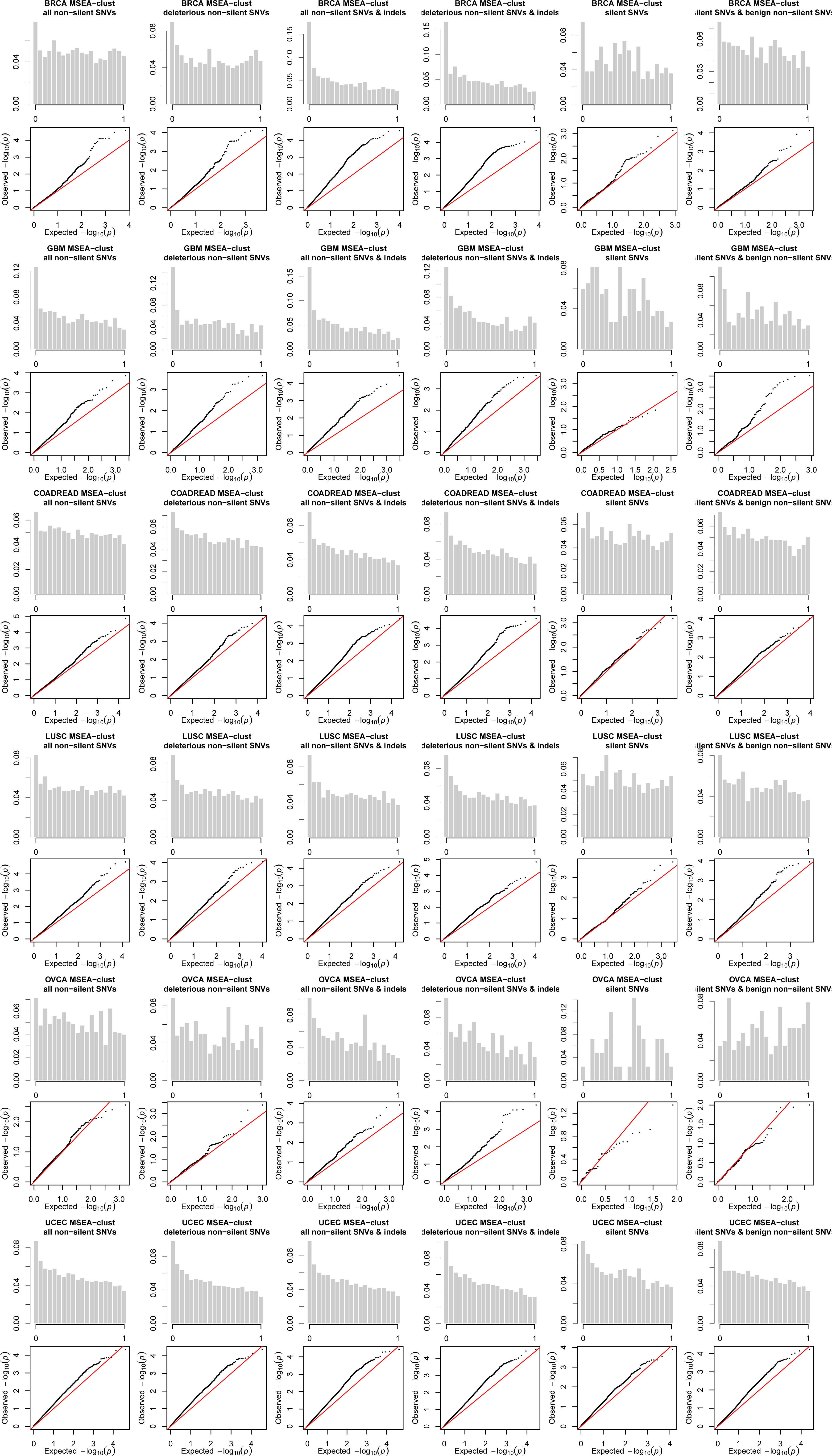
Figure S1. Histograms and Q-Q plot of *p*-values obtained by MSEA-clust for each cancer using different mutations. For each cancer, *p-*values obtained using six mutation sets were plotted: all non-silent SNVs, deleterious non-silent SNVs, all non-silent SNVs plus indels, deleterious non-silent SNVs plus indels, all silent (synonymous) SNVs, and all silent SNVs plus benign non-silent SNVs. In the histograms, x-axis denotes *p-*value and y-axis denotes the proportion of genes in each *p-*value interval. In the Q-Q plot, the x-axis is the expected *p*-value and the y-axis is the observed *p*-value. The red line indicates the reference line y=x.


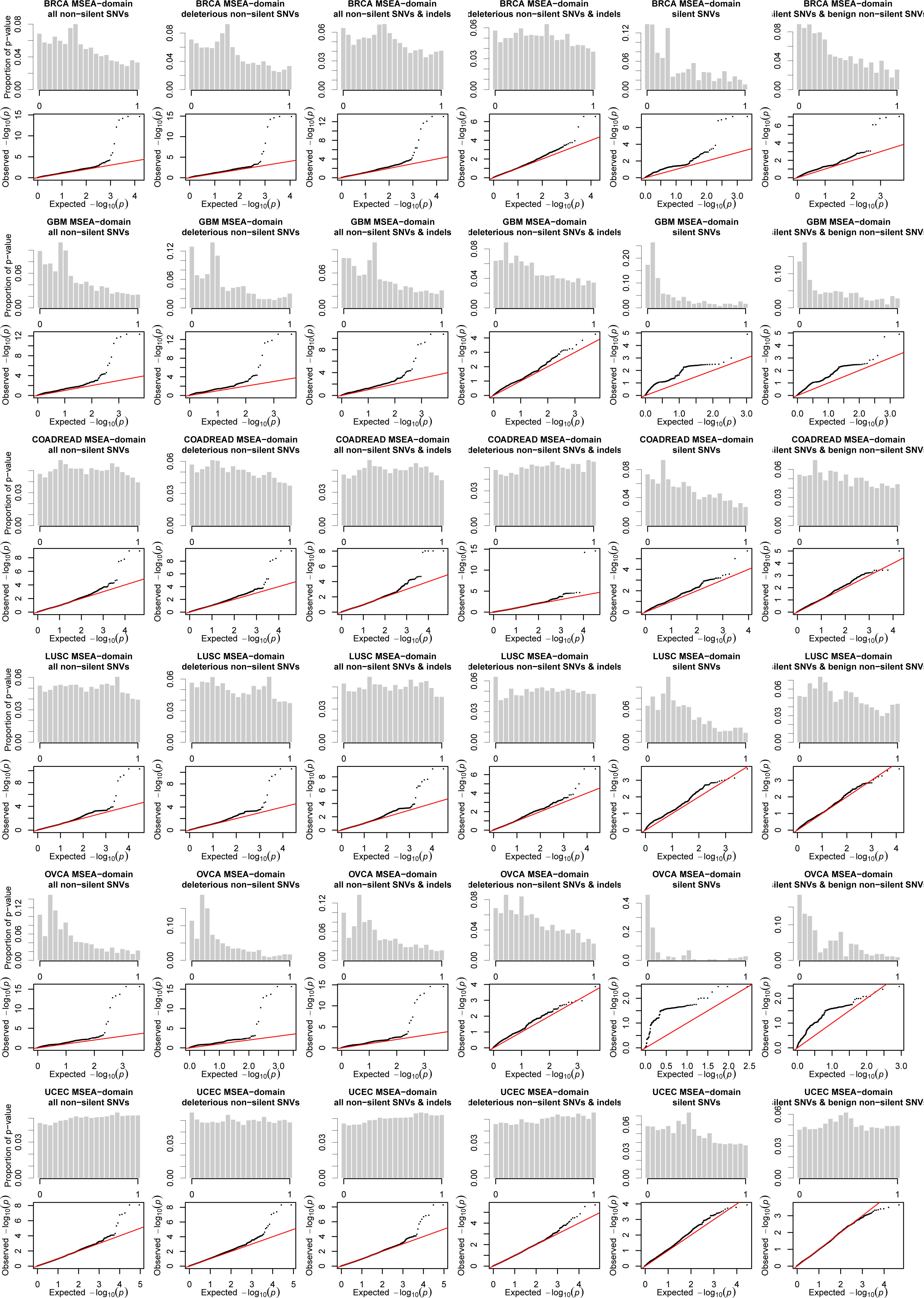
Figure S2. Q-Q plot of *p-*values obtained by MSEA-domain (M1) for each cancer using different mutations (see legend of Figure S1).


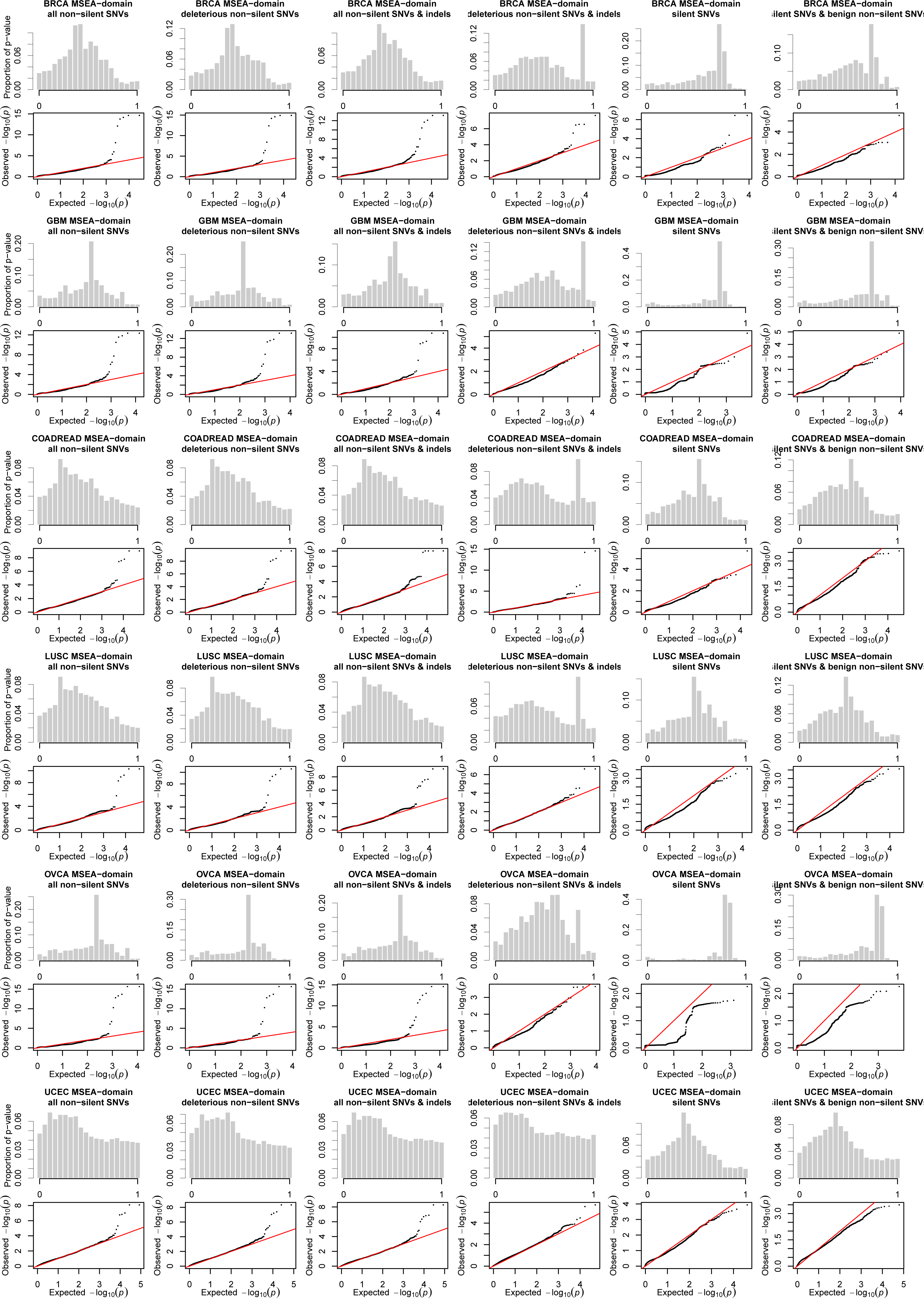


Figure S3. Q-Q plot of *p-*values obtained by MSEA-domain (M2) for each cancer using different mutations.


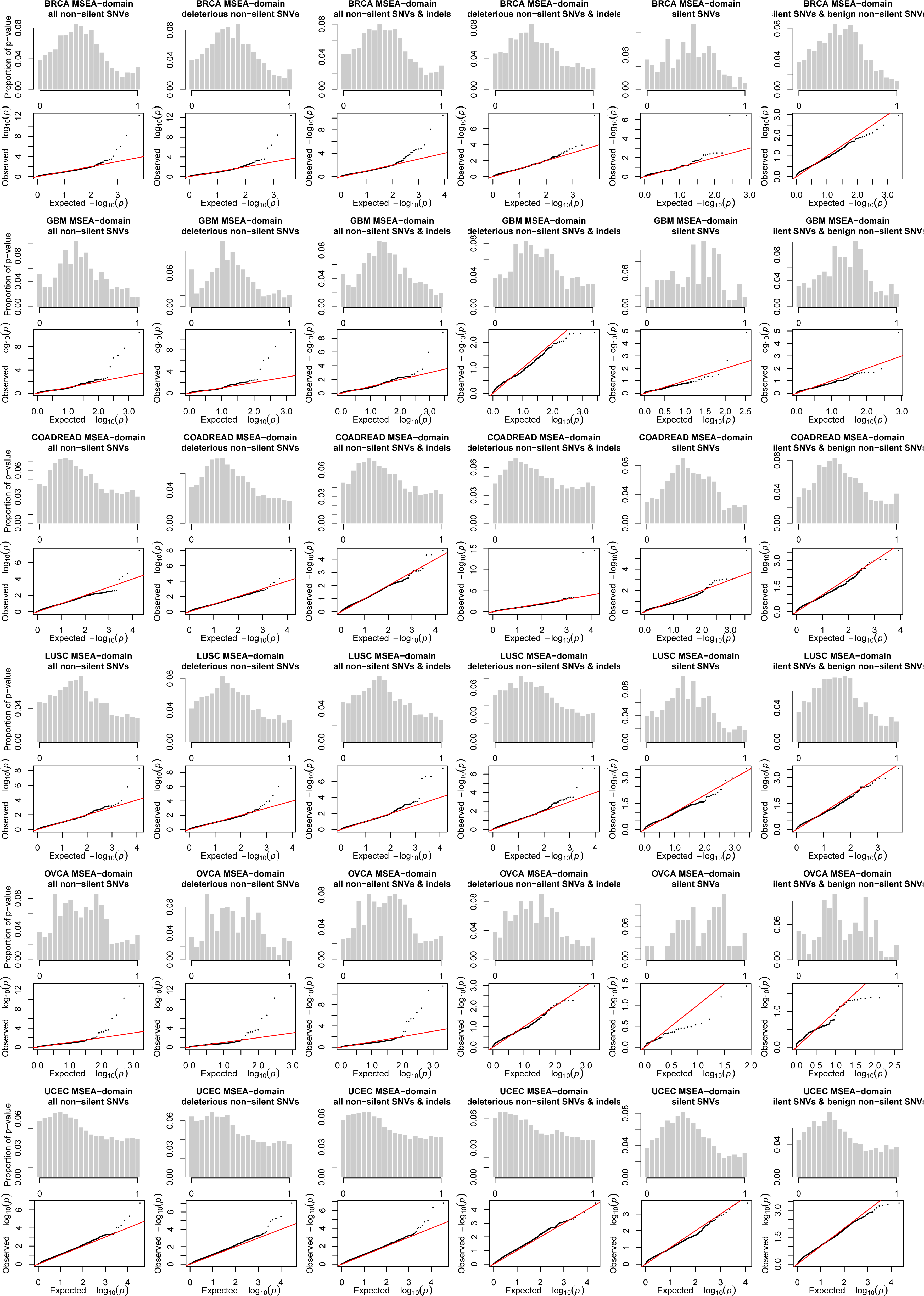
Figure S4. Q-Q plot of *p-*values obtained by MSEA-domain (M3) for each cancer using different mutations.


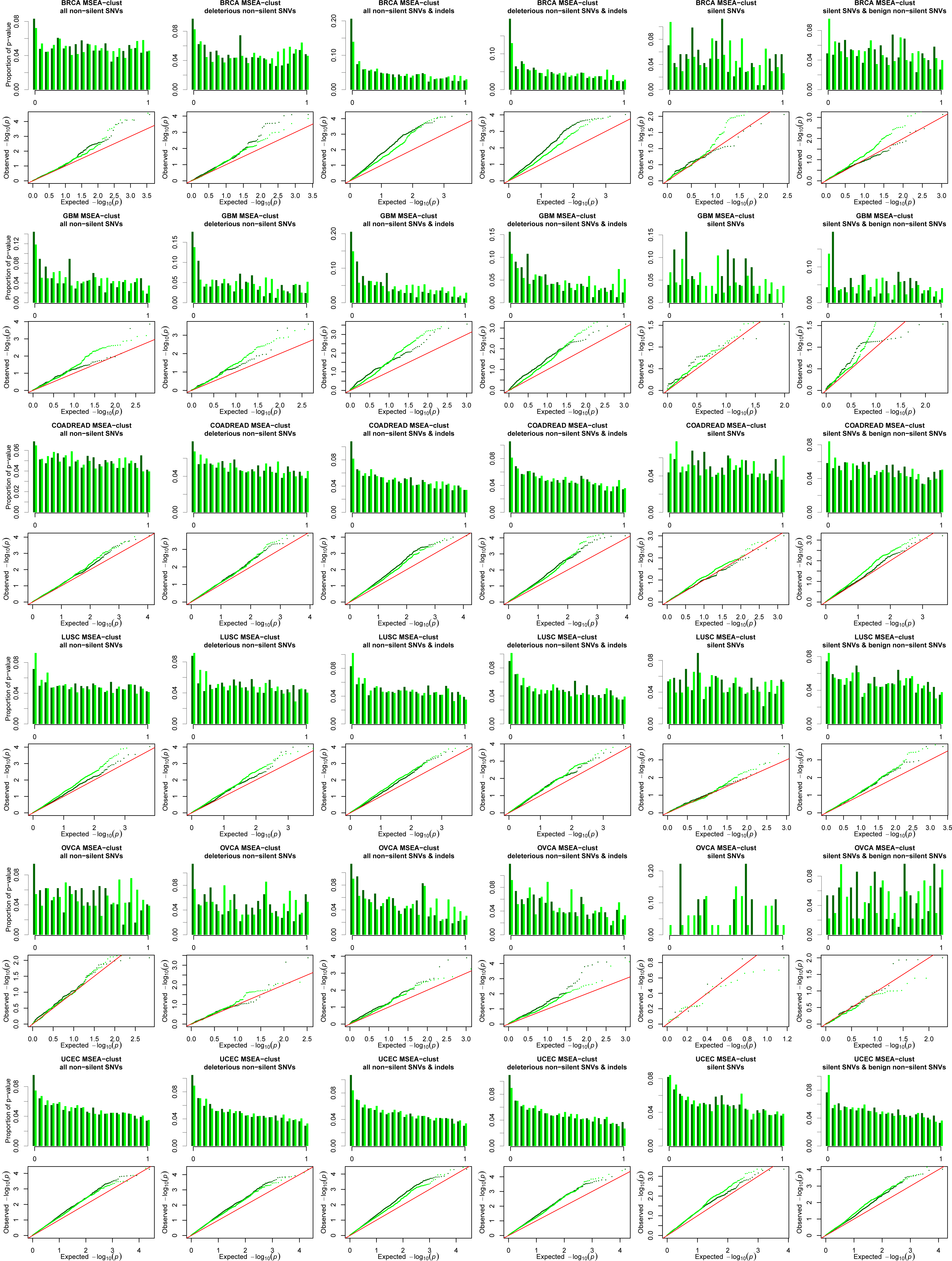


Figure S5. Q-Q plot of *p-*values obtained by MSEA-clust for each cancer using different mutations in expressed (dark green) and unexpressed (light green) genes.


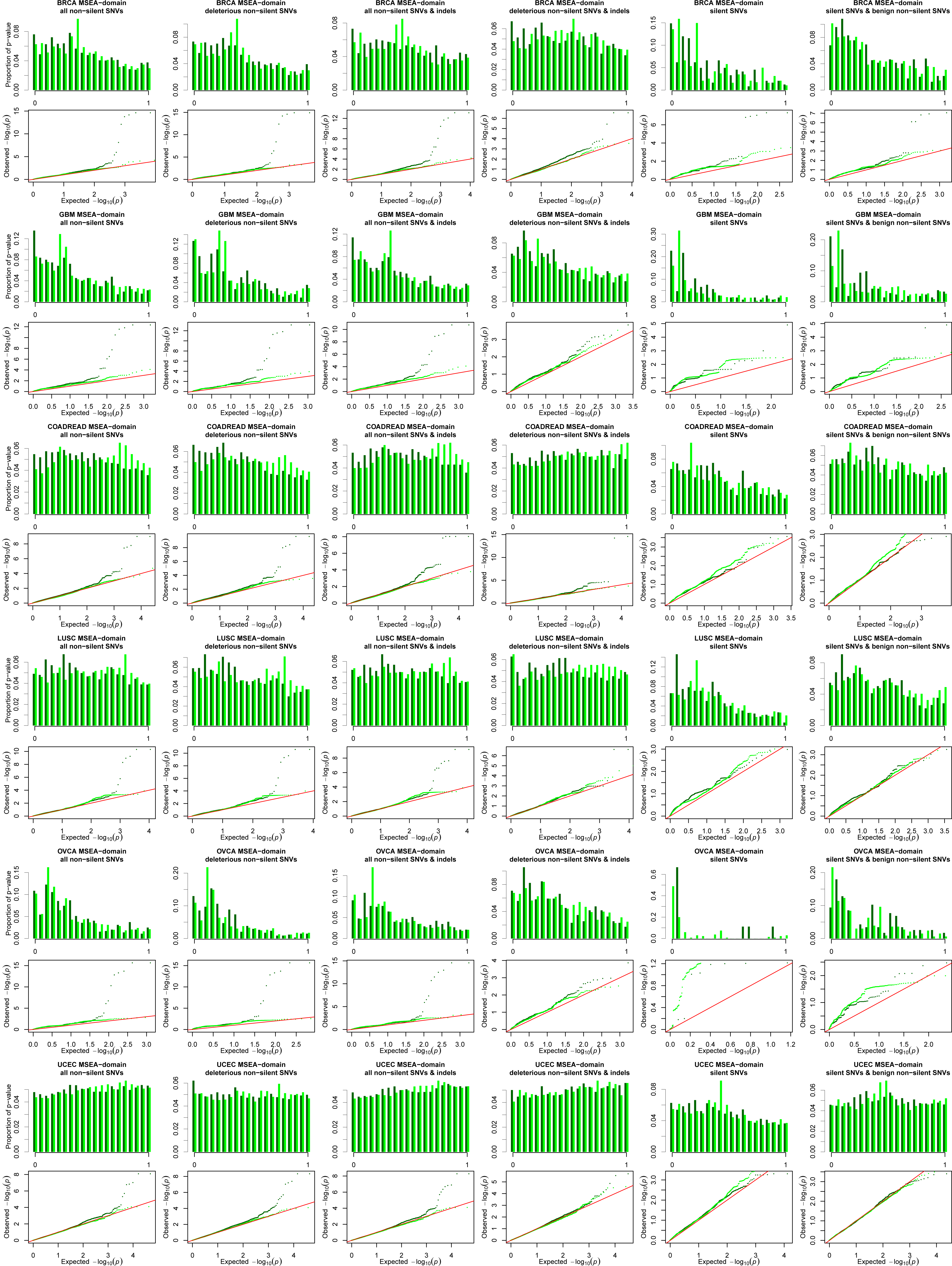


Figure S6. Q-Q plot of *p-*values obtained by MSEA-domain (M1) for each cancer using different mutations in expressed (dark green) and unexpressed (light green) genes.


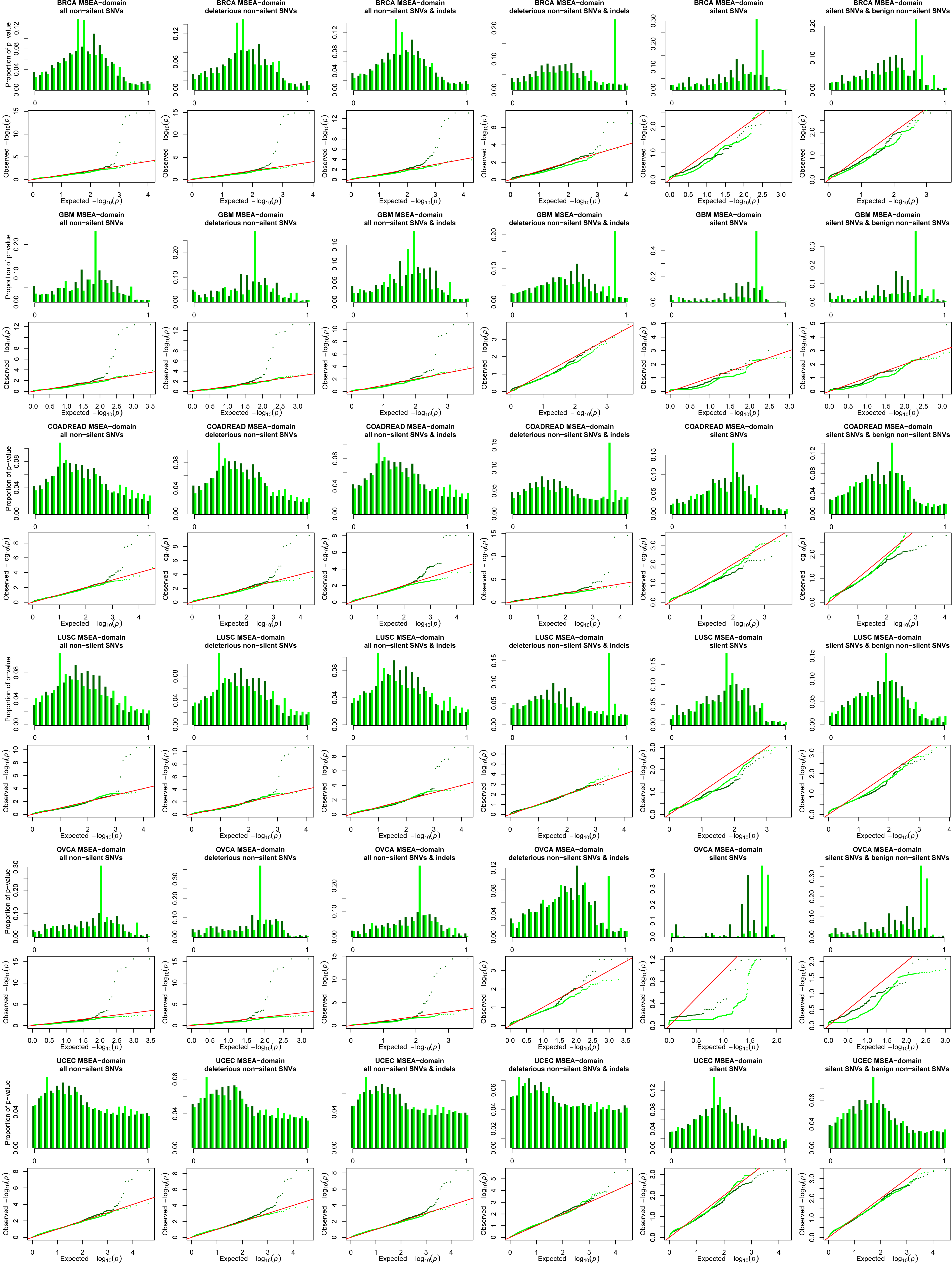


Figure S7. Q-Q plot of *p-*values obtained by MSEA-domain (M2) for each cancer using different mutations in expressed (dark green) and unexpressed (light green) genes.


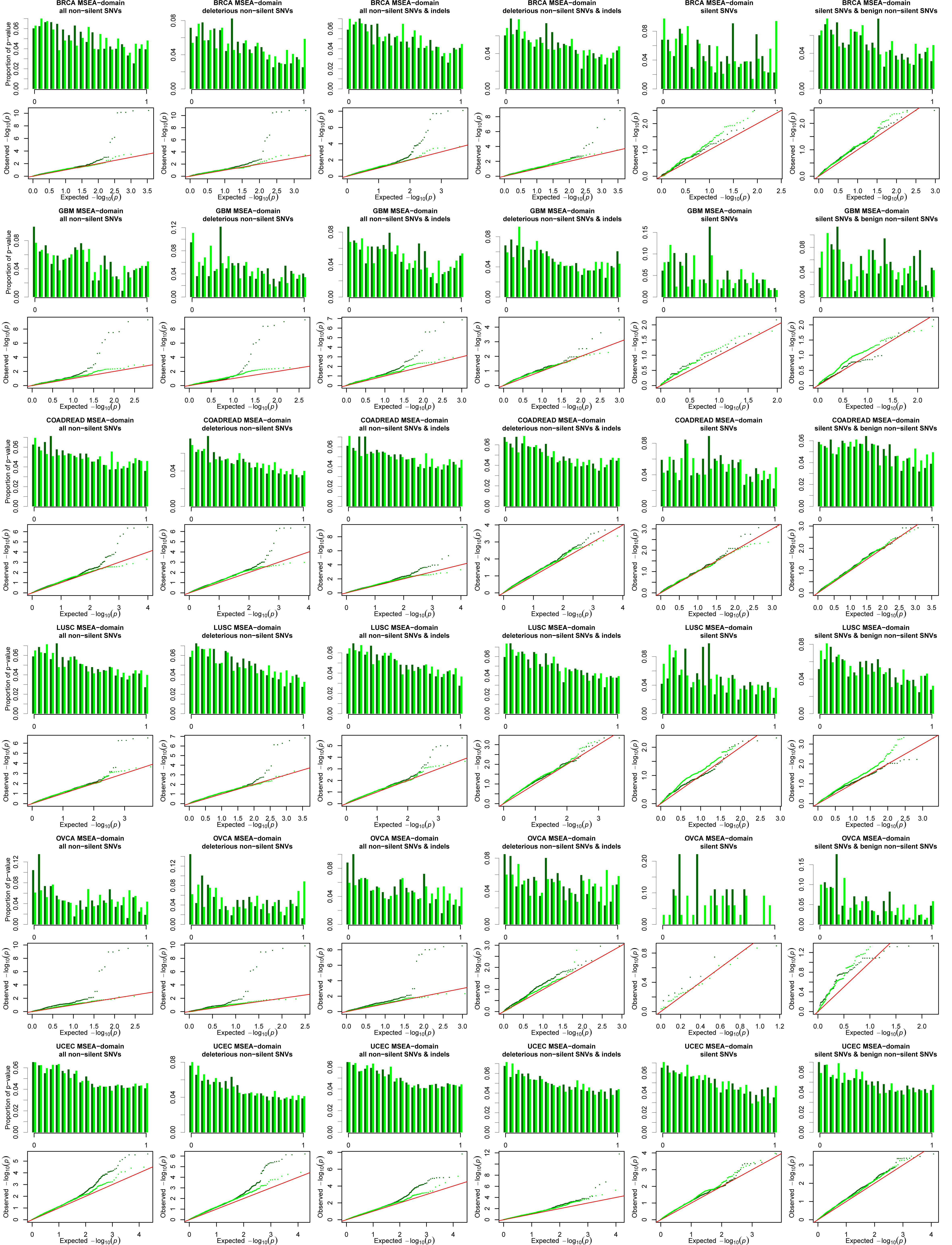


Figure S8. Q-Q plot of *p-*values obtained by MSEA-domain (M3) for each cancer using different mutations in expressed (dark green) and unexpressed (light green) genes.


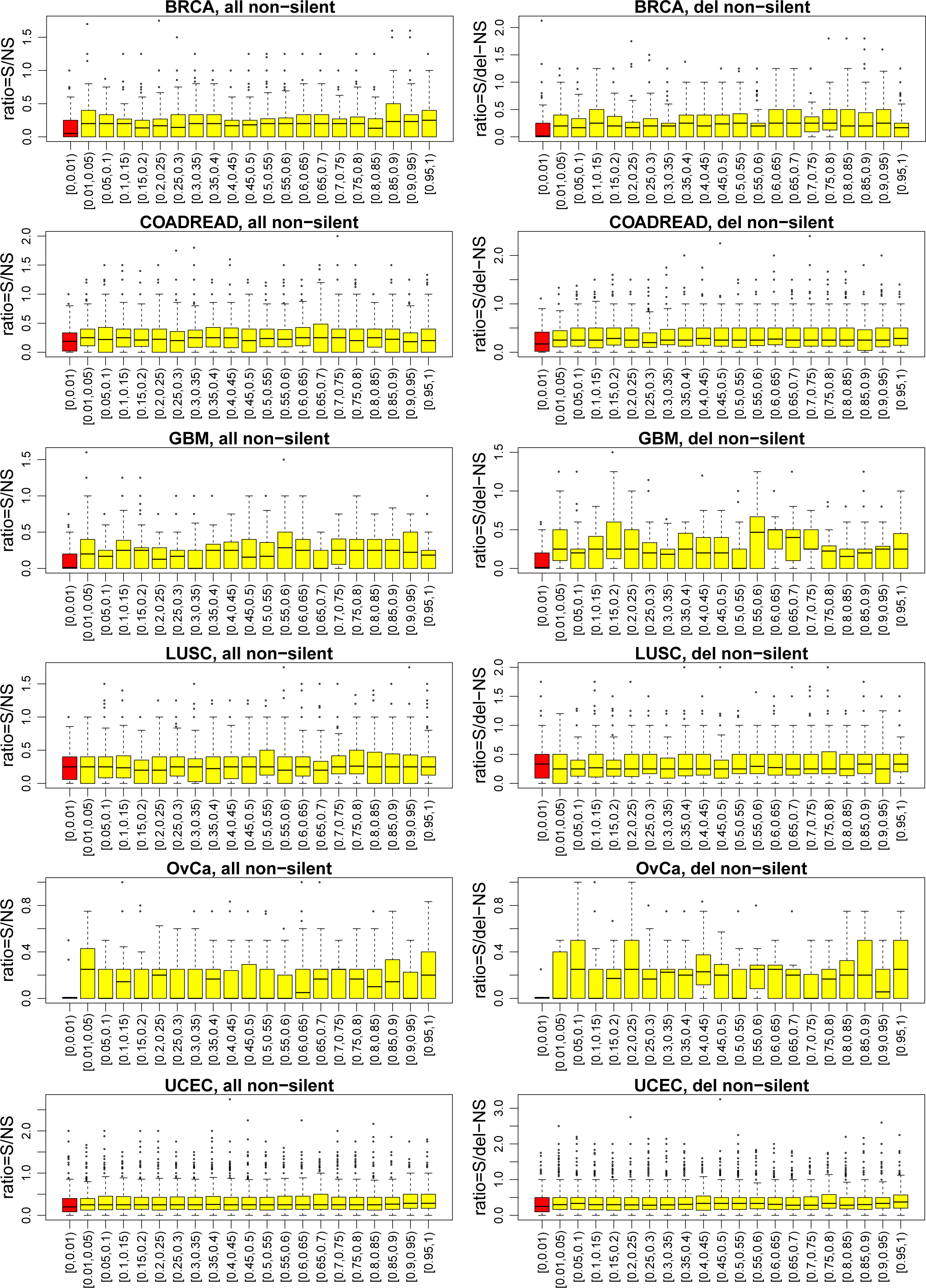


Figure S9. The ratio of silent SNVs vs. non-silent SNVs in each cancer. x-axis: *p*-value. y-axis: the *S/NS* ratio, i.e., $S/NS=\frac{\#silent SNVs}{\#non-silent SNVs}$. Del: deleterious.


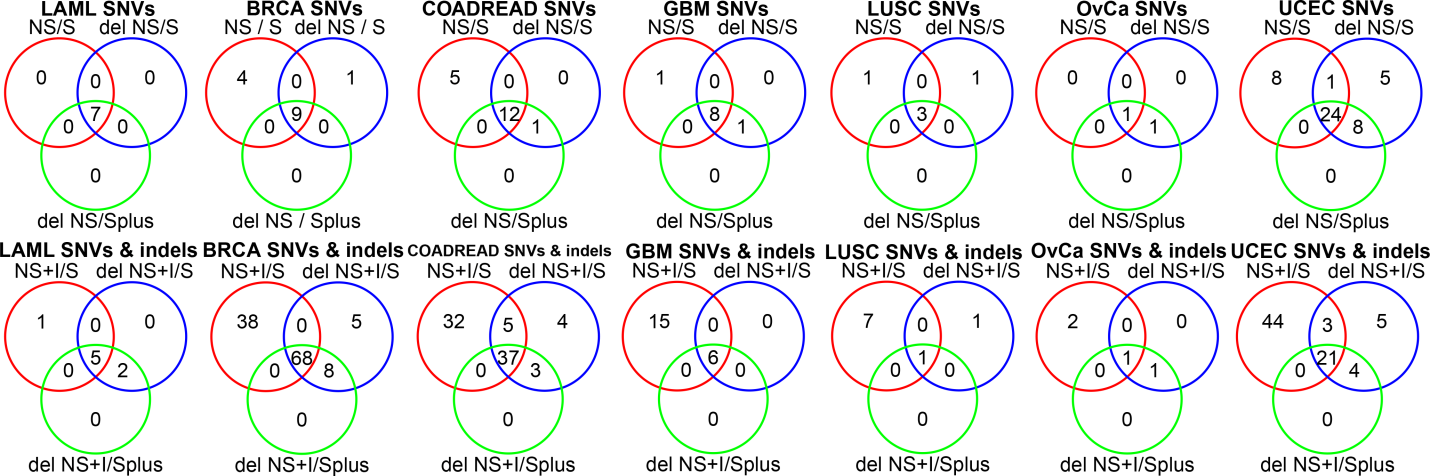

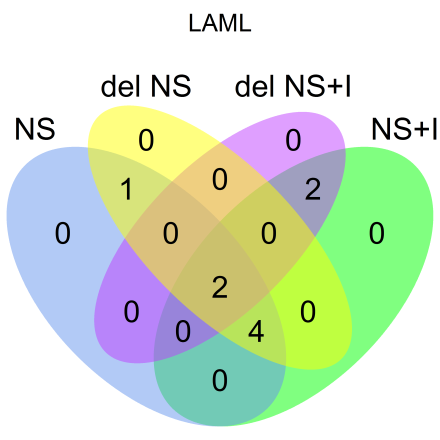

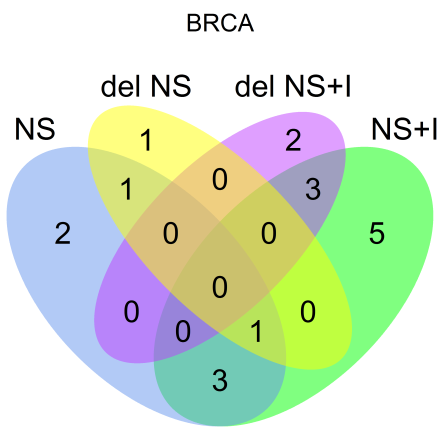

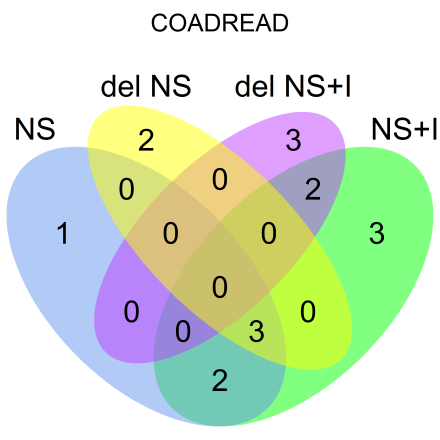


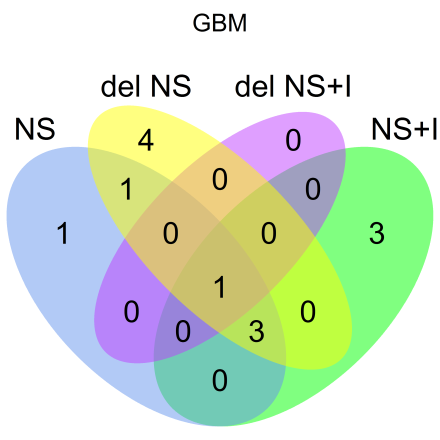

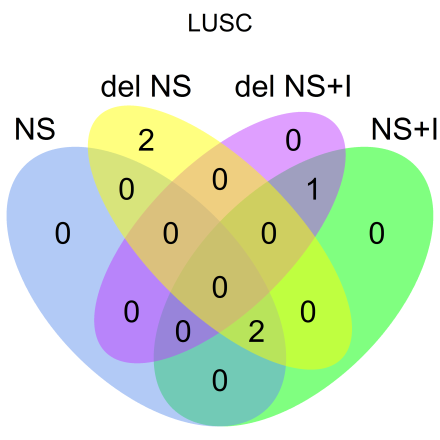

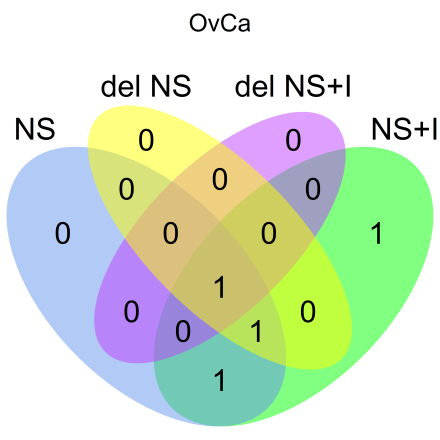


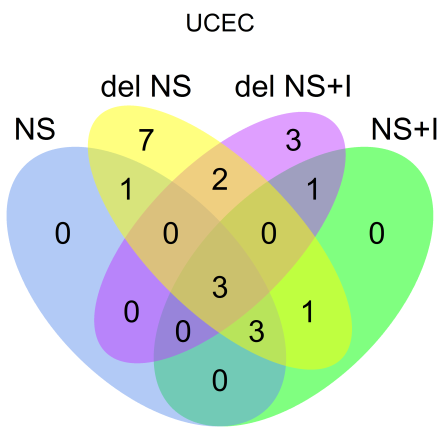


Figure S10. Results comparison between different models. (A) MSEA-clust results in each cancer type. Top panel presents comparion among genes obtained using SNVs only in each of the following scenario: (1) non-silent SNVs vs. the background formed by silent SNVs (NS/S), (2) deleterious non-silent SNVs vs. silent SNVs (del NS/S), (3) deleterious non-silent SNVs vs. silent plus benign missense SNVs (del NS/Splus). Bottom panel presents comparison among genes obtained using SNVs plus indels in each of the following scenarios: (4) non-silent SNVs plus indels vs. silent SNVs (NS+I/S), (5) deleterious non-silent SNVs plus indels vs. silent SNVs (del NS+I/S), and (6) deleterious non-silent SNVs plus indels vs. silent plus benign missense SNVs (del NS+I/Splus). (B) MSEA-domain results in each cancer type. The genes obtained in each of the four scenarios were compared: (1) non-silent SNVs (NS), (2) deleterious non-silent SNVs (del NS), (3) non-silent SNVs plus indels (NS+I), and (4) deleterious non-silent SNVs plus indels (del NS+I).


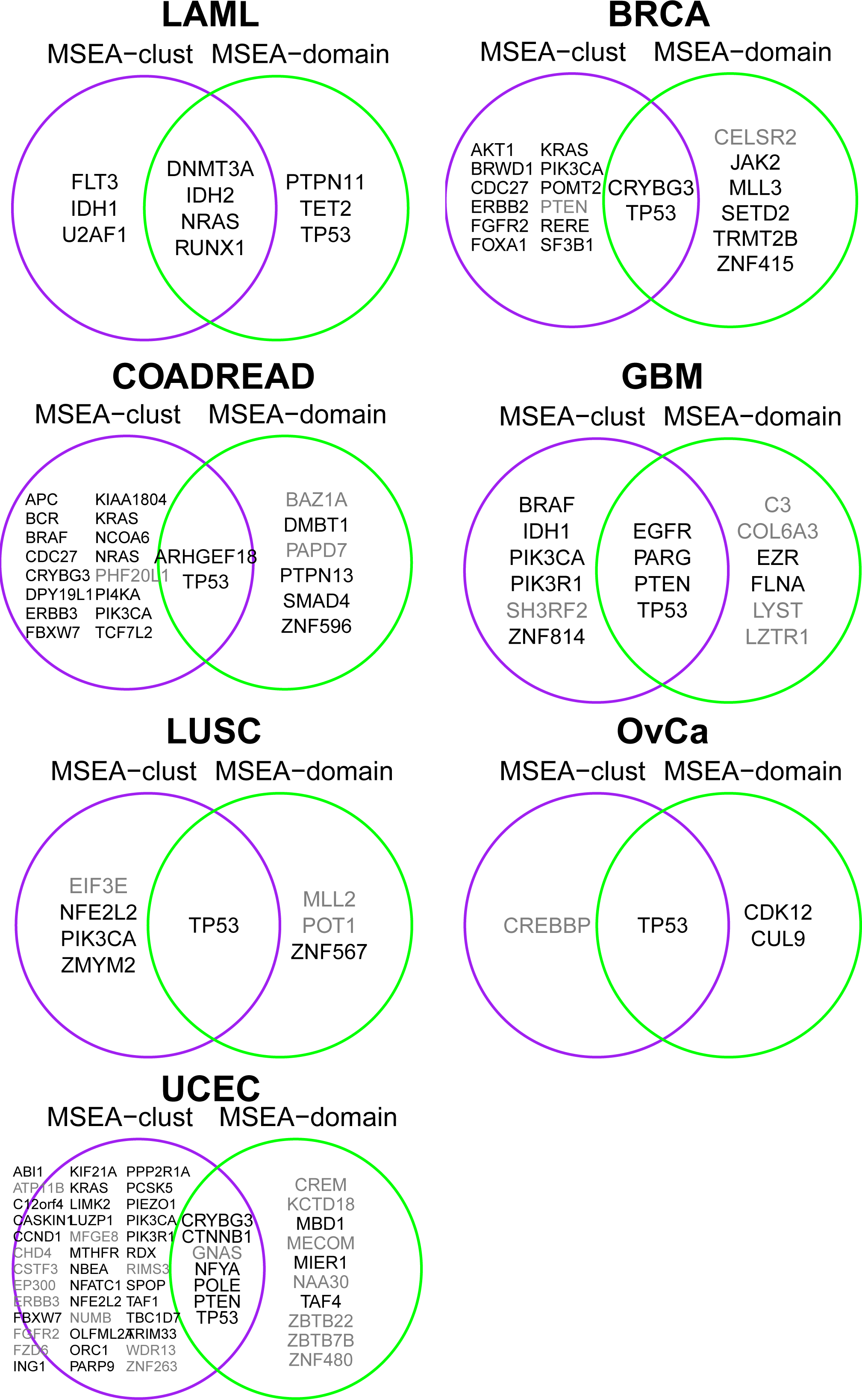


Figure S11. Comparison of significant genes by MSEA-clust and MSEA-domain in each cancer using SNVs only. Genes in grey were only detected when using all non-silent SNVs. Genes in black were detected both when using all non-silent SNVs and when using deleterious non-silent SNVs.


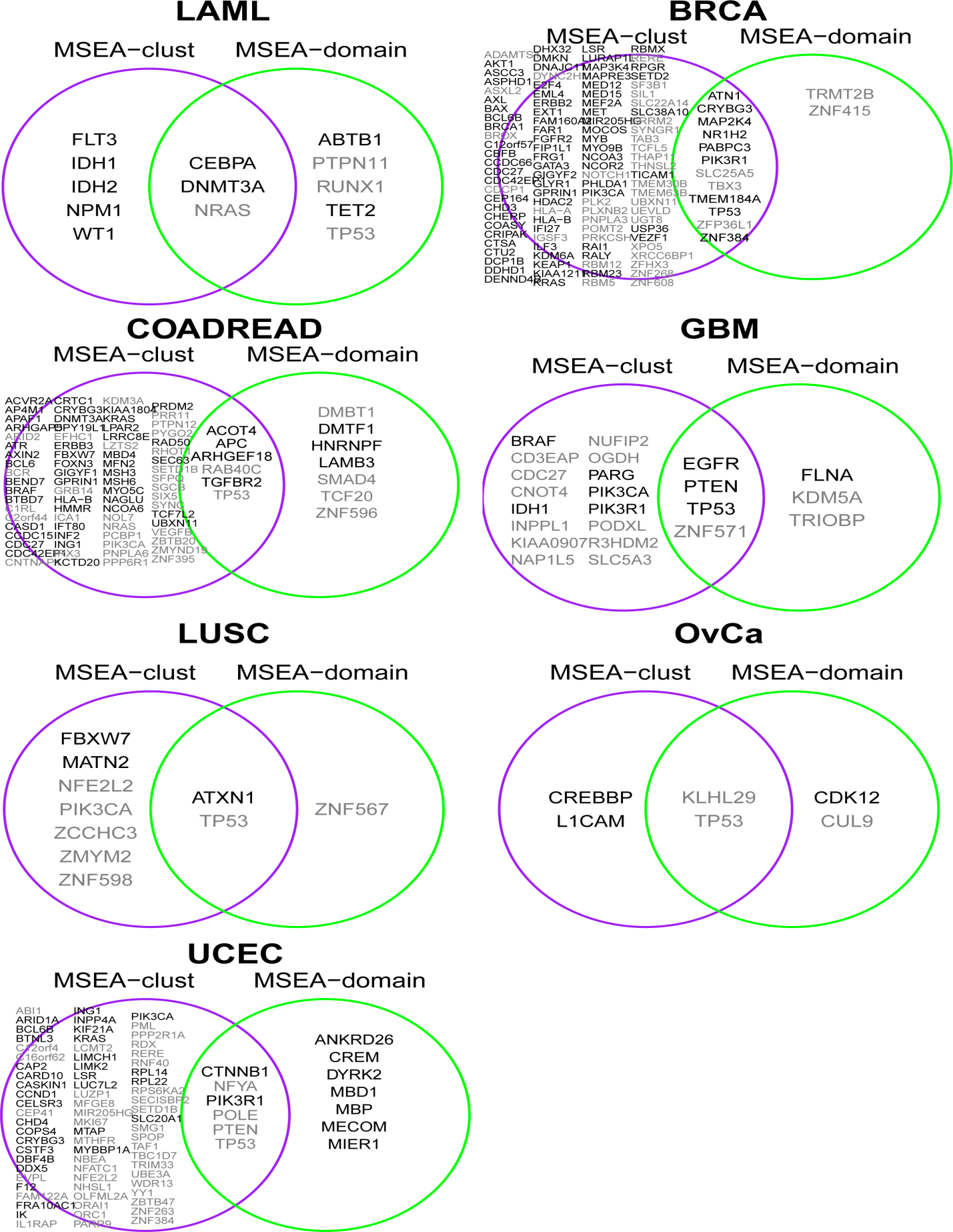


Figure S12. Comparison of significant genes by MSEA-clust and MSEA-domain in each cancer using SNVs and indels. Genes in grey were only detected when using all non-silent SNVs. Genes in black were detected both when using all non-silent SNVs and when using deleterious non-silent SNVs.


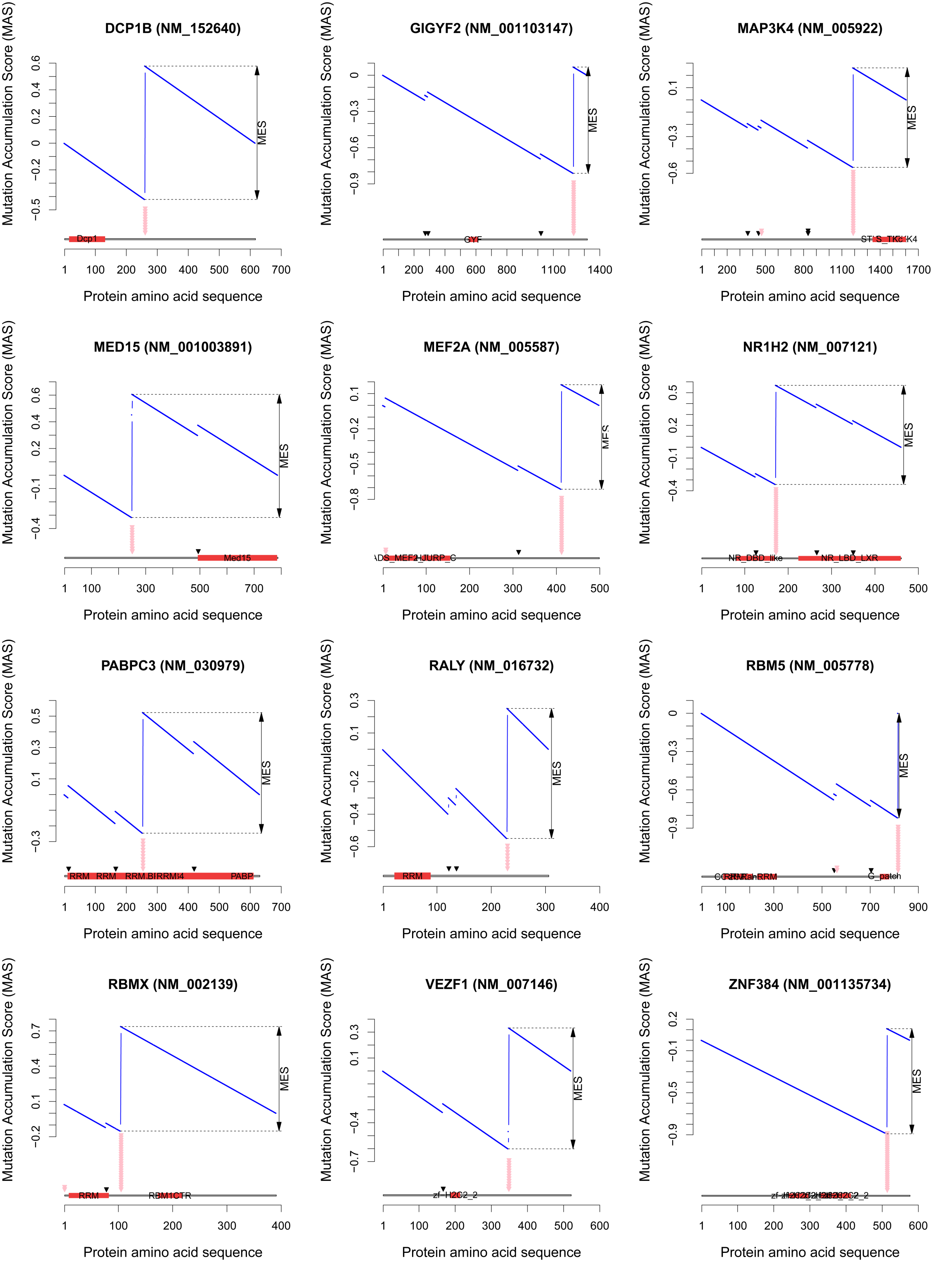
Figure S13. Genes of interest in BRCA that were uniquely detected when including indels. Pink triangle indicates indels. Due to space limitation, we only draw genes with ≥10 mutations (SNVs and indels).


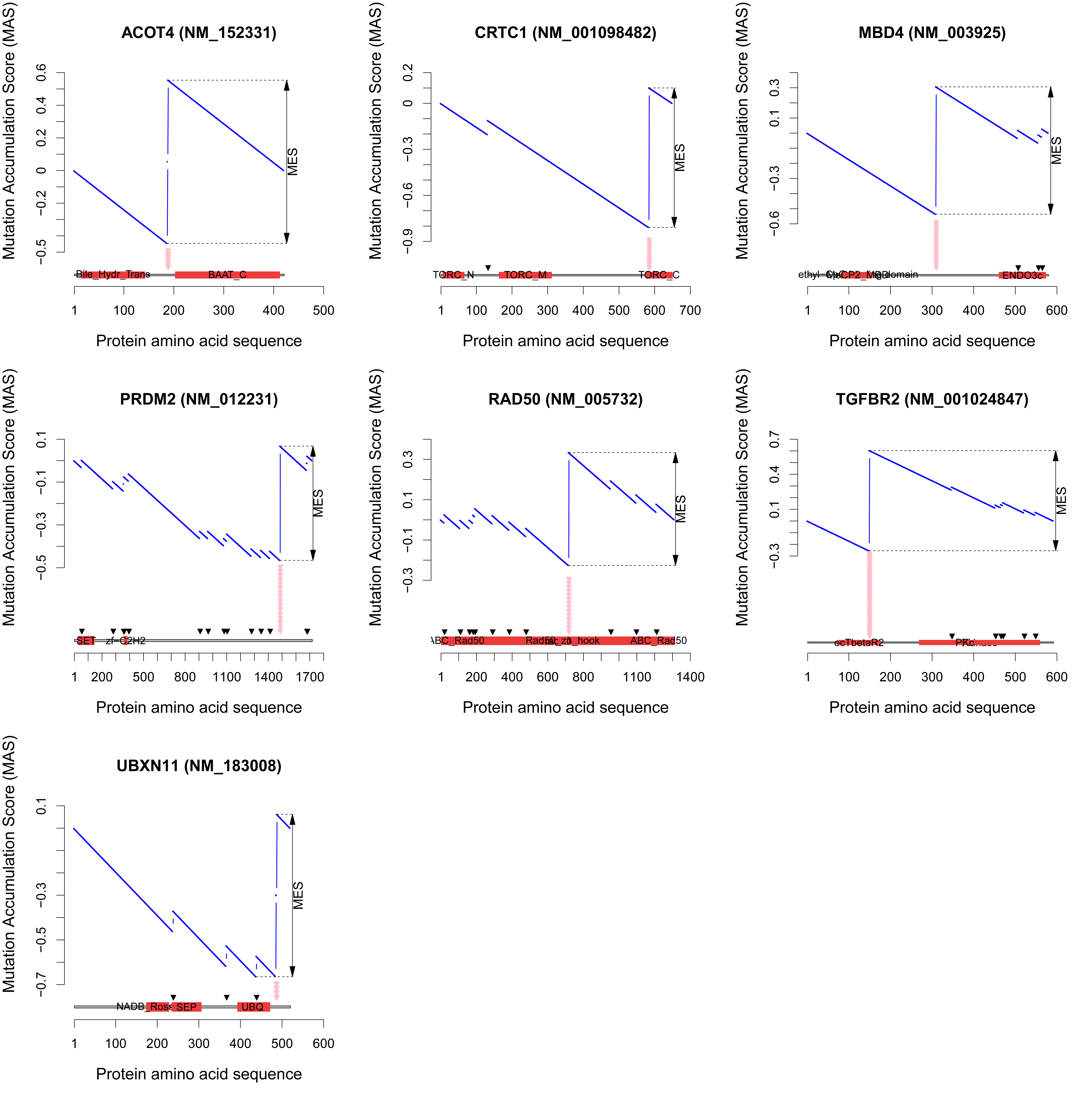
Figure S14. Genes of interest (peak within 3 amino acids) in COADREAD that were uniquely detected when including indels. Pink triangle indicates indels. Due to space limitation, we only draw genes with ≥10 mutations (SNVs and indels) and with domain annotations.


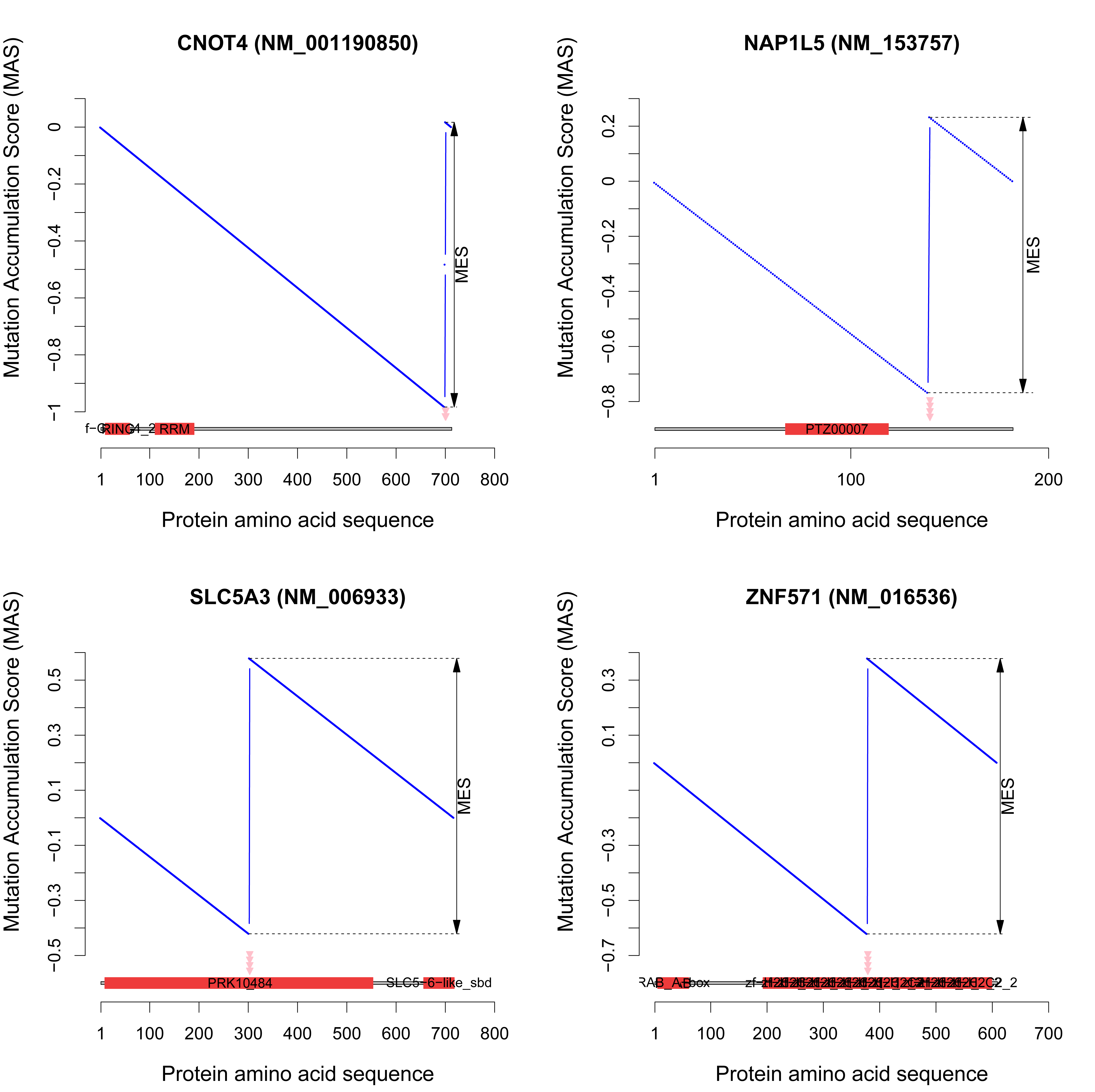
Figure S15. Genes of interest (peak within 3 amino acids) in GBM that were uniquely detected when including indels. Pink triangle indicates indels. Due to space limitation, we only draw genes with ≥10 mutations (SNVs and indels) and with domain annotations.


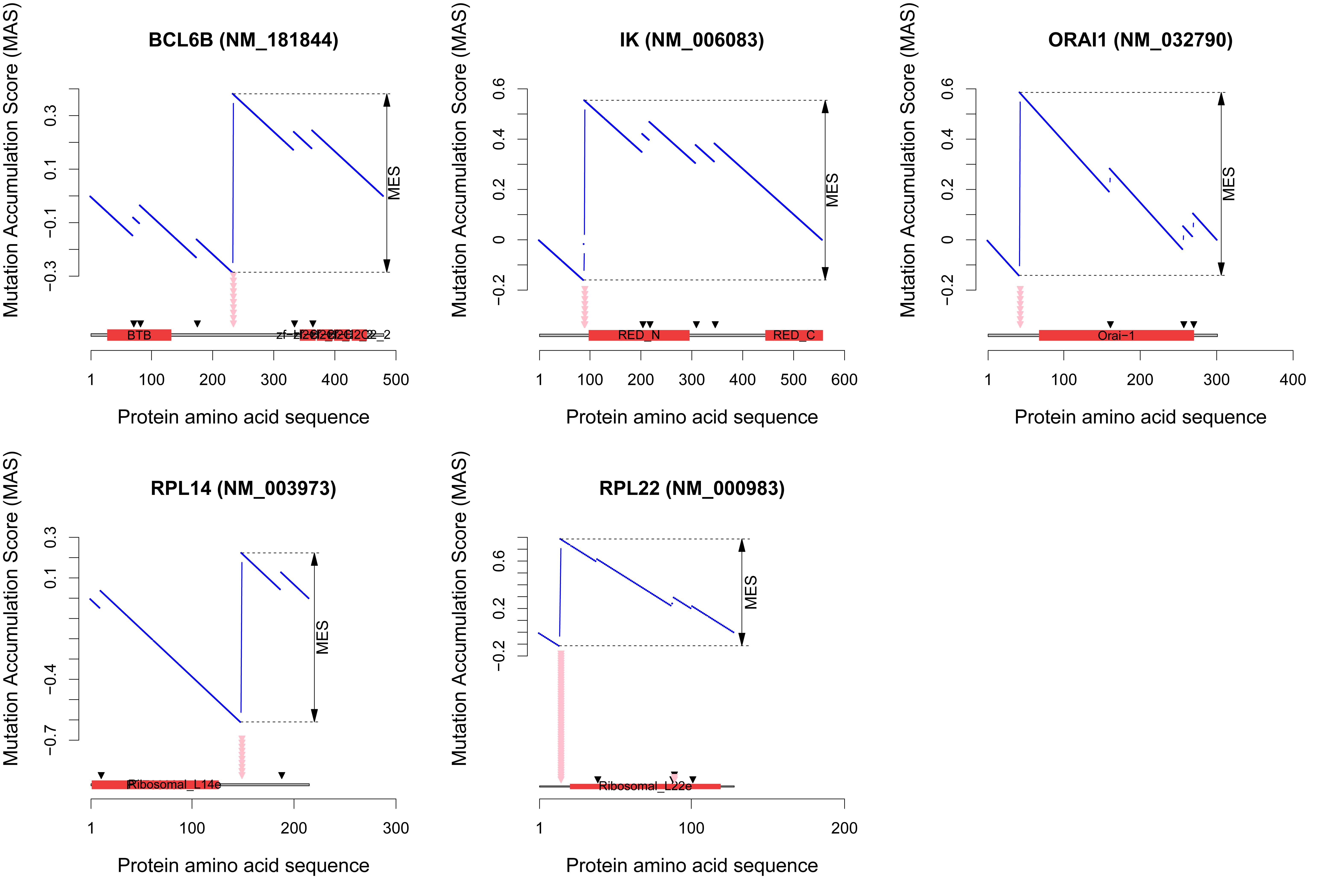


Figure S16. Genes of interest (peak within 3 amino acids) in UCEC that were uniquely detected when including indels. Pink triangle indicates indels. Due to space limitation, we only draw genes with ≥10 mutations (SNVs and indels) and with domain annotations.


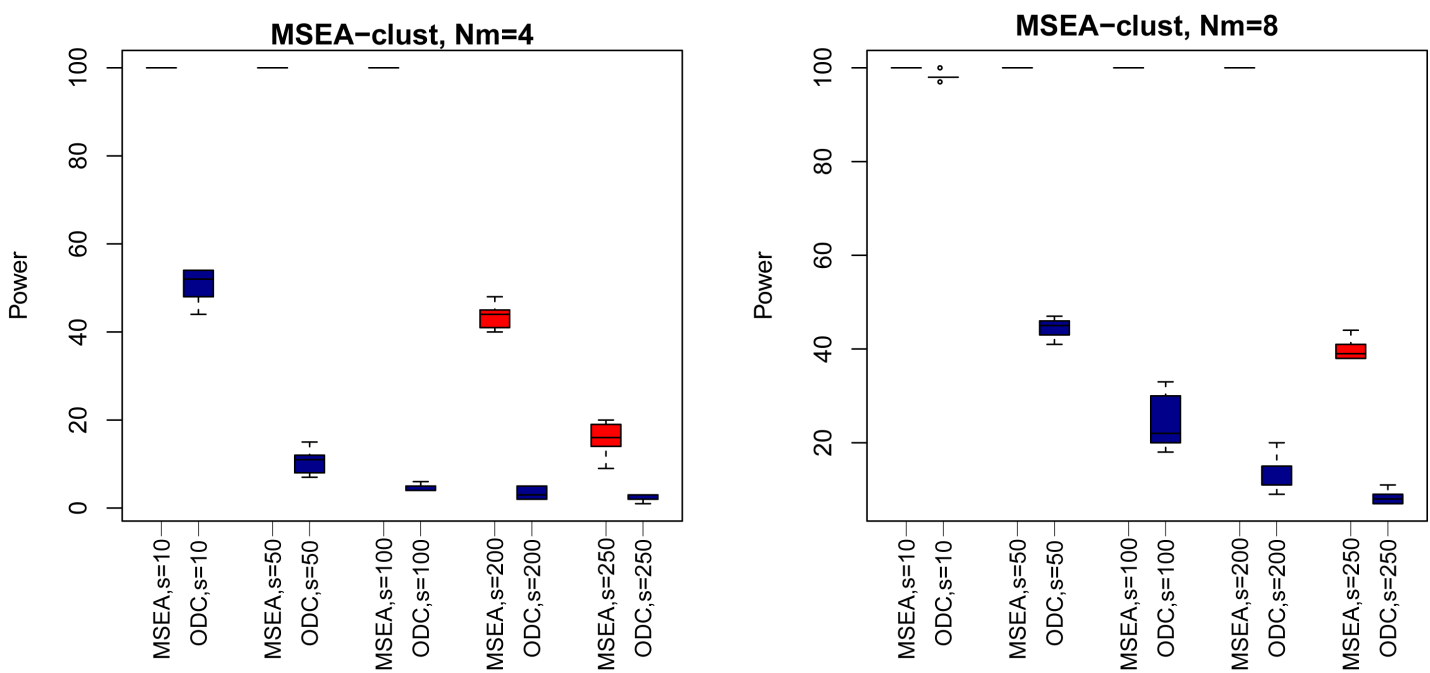


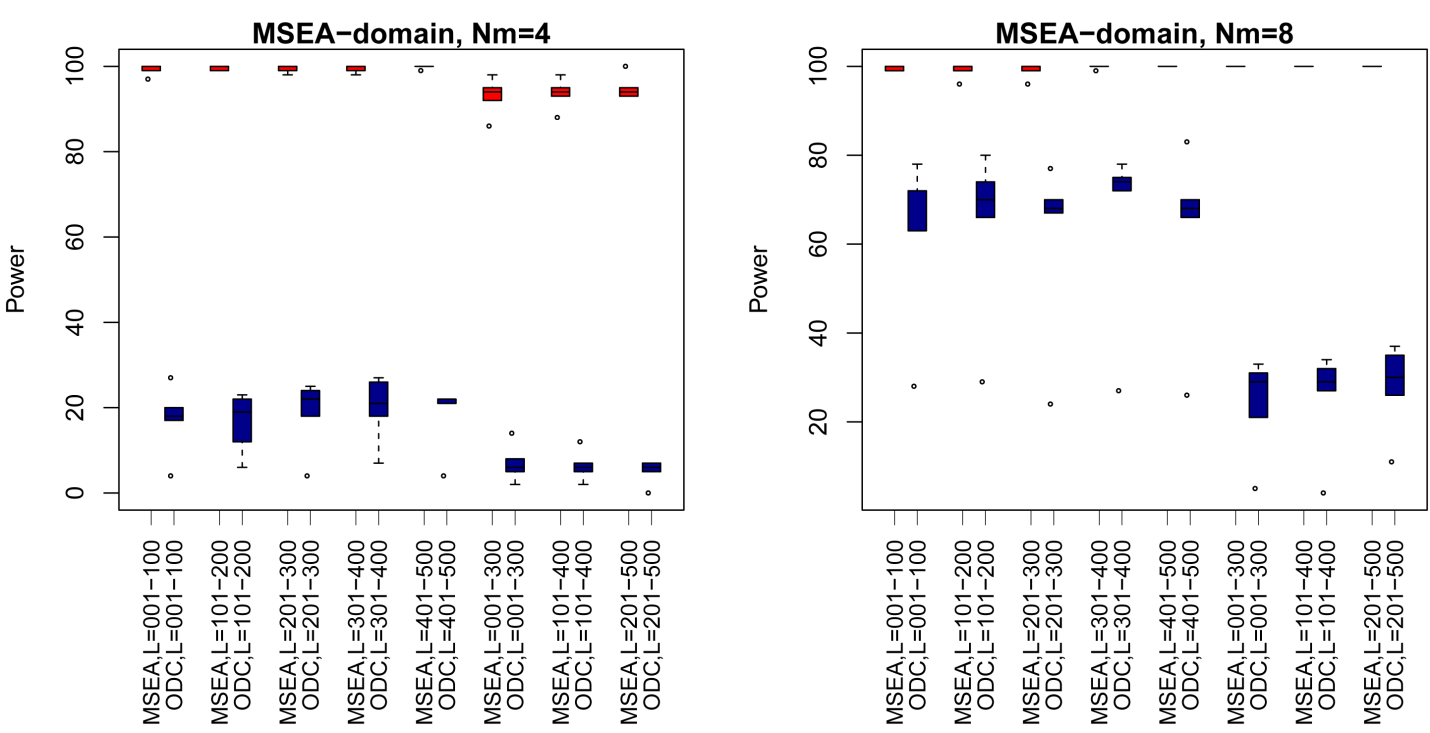


Figure S17. Power estimation of MSEA and OncodriveCLUST in different scenarios. Top panels: MSEA-clust vs. OncodriveCLUST; bottom panels: MSEA-domain vs. OncodriveCLUST. Red box: MSEA; blue box: OncodriveCLUST (ODC). In the x-axis, s denotes spanning regions (top panels), and L denotes domain location (bottom panel). All results are based on the same simulation data as used in Table S2 with recurrent mutations allowed.


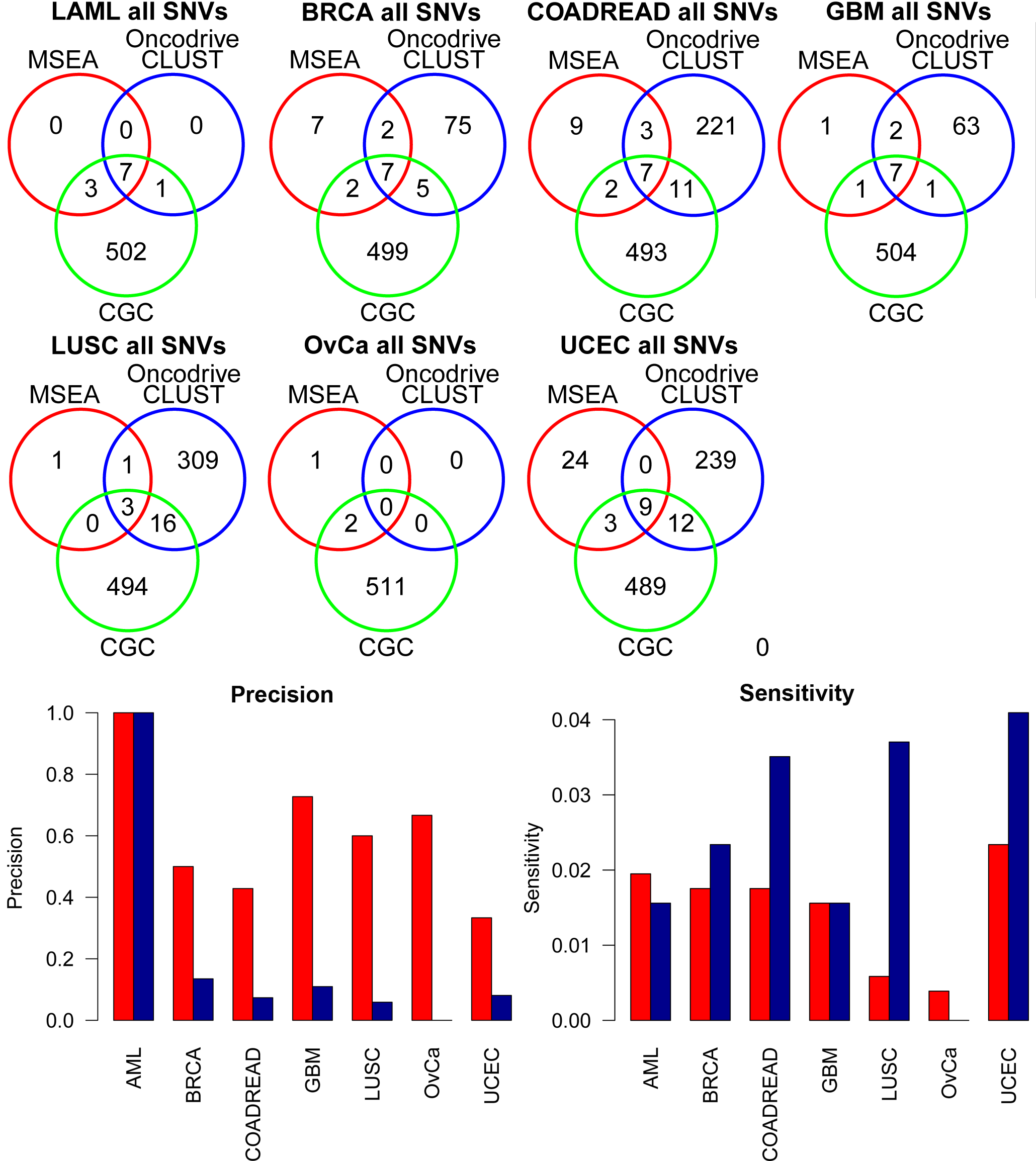


Figure S18. Venn diagram of significant genes identified by MSEA and OncodriveCLUST in comparison with CGC genes. These results were obtained using all non-silent SNVs. Red bar: MSEA; blue bar: OncodriveCLUST.


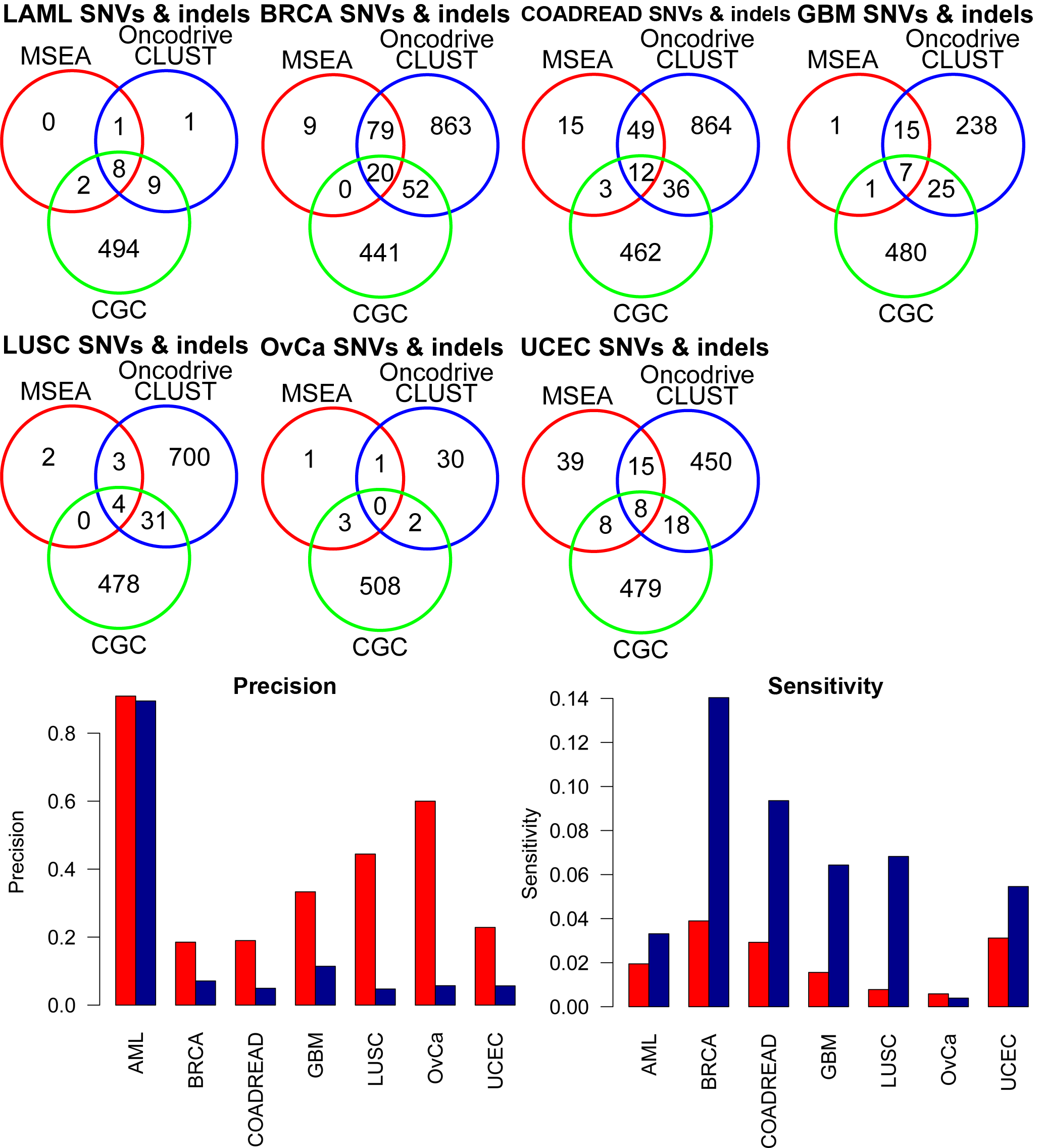


Figure S19. Venn diagram of significant genes identified by MSEA and OncodriveCLUST in comparison with CGC genes. These results were obtained using all non-silent SNVs plus indels. Red bar: MSEA; blue bar: OncodriveCLUST.


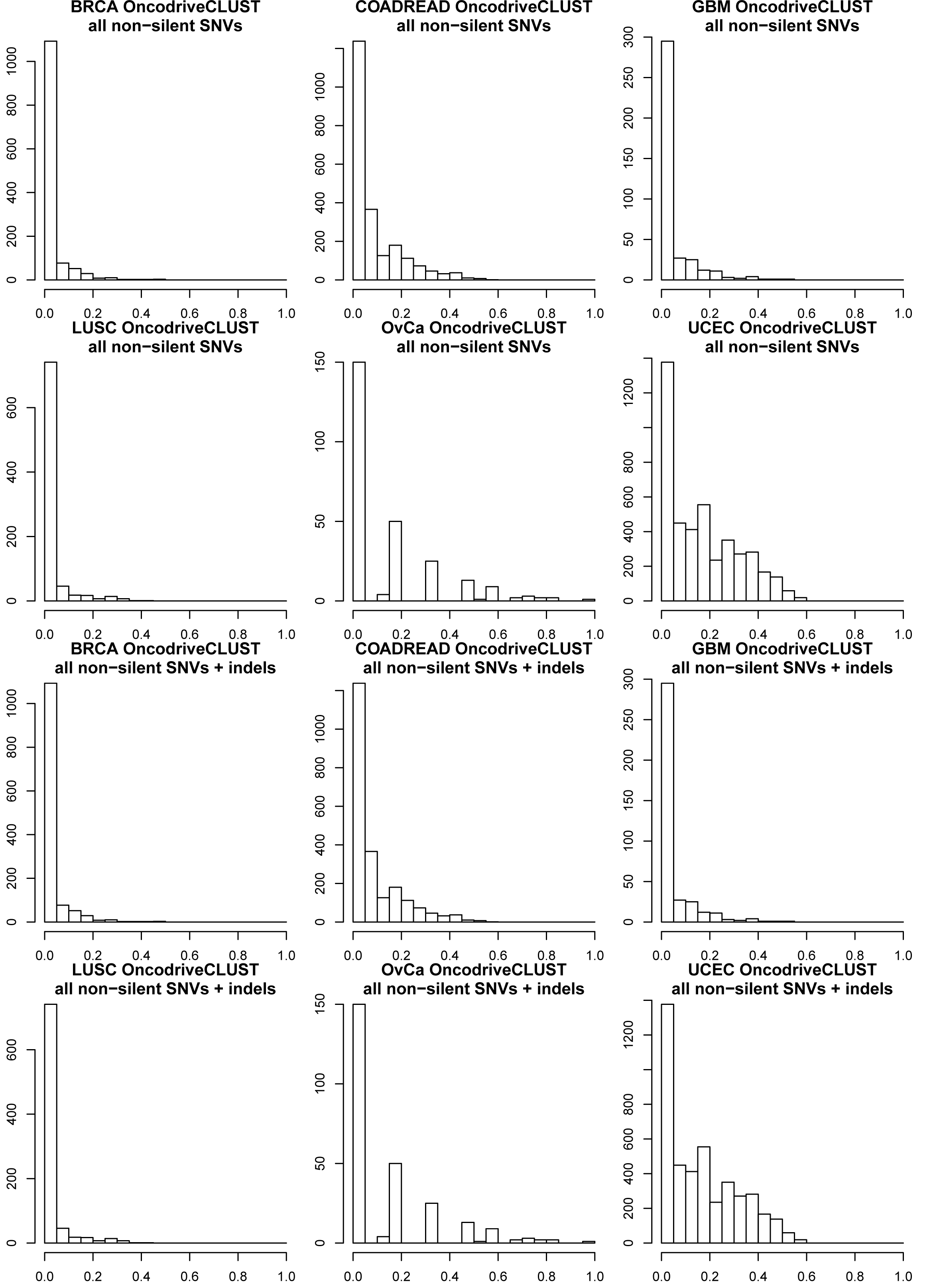


Figure S20. Histograms of gene-based *p-*values by OncodriveCLUST for each cancer.
